# Direct identification of bacterial and human proteins from infected wounds in living 3D skin models

**DOI:** 10.1038/s41598-020-68233-6

**Published:** 2020-07-17

**Authors:** Jana Havlikova, Robin C. May, Iain B. Styles, Helen J. Cooper

**Affiliations:** 1 EPSRC Centre for Doctoral Training in Physical Sciences for Health, University of Birmingham, Edgbaston, Birmingham, B15 2TT UK; 2 School of Biosciences, University of Birmingham, Edgbaston, Birmingham, B15 2TT UK; 3 Institute of Microbiology and Infection, University of Birmingham, Edgbaston, Birmingham, B15 2TT UK; 4 School of Computer Science, University of Birmingham, Edgbaston, Birmingham, B15 2TT UK; 5 Centre of Membrane Proteins and Receptors, The Universities of Birmingham and Nottingham, The Midlands, Birmingham, UK; 6 Alan Turing Institute, 96 Euston Road, London, NW1 2DB UK

**Keywords:** Analytical chemistry, Microbiology, Infectious diseases

## Abstract

Trauma is one of the leading causes of death in people under the age of 49 and complications due to wound infection are the primary cause of death in the first few days after injury. The ESKAPE pathogens are a group of bacteria that are a leading cause of hospital-acquired infections and a major concern in terms of antibiotic resistance. Here, we demonstrate a novel and highly accurate approach for the rapid identification of ESKAPE pathogens (***Enterococcus faecium, Staphylococcus aureus, Klebsiella pneumoniae***, ***Acinetobacter baumannii***, ***Pseudomonas aeruginosa,*** and ***Enterobacter*** spp.) directly from infected wounds in 3D in vitro skin models. Wounded skin models were inoculated with bacteria and left to incubate. Bacterial proteins were identified within minutes, directly from the wound, by liquid extraction surface analysis mass spectrometry. This approach was able to distinguish closely related strains and, unlike genomic approaches, can be modified to provide dynamic information about pathogen behaviour at the wound site. In addition, since human skin proteins were also identified, this method offers the opportunity to analyse both host and pathogen biomarkers during wound infection in near real-time.

## Introduction

Trauma is one of the leading causes of death in people under the age of 49^1^. Complications due to infection are the primary cause of death in patients who survive the first few days after a traumatic injury. In recent military conflicts, over half of the total injuries sustained were the result of trauma to the extremities^2^. Over 25% of those patients suffered complications due to infection (rising to 50% for those requiring intensive care), either in the bones (osteomyelitis) or deep-wound infection^2,3^. Similar rates of infection are associated with civilian trauma^2^. Infectious complications result in significantly higher amputation rates, which are also of greater severity. The ESKAPE pathogens (***Enterococcus faecium, Staphylococcus aureus, Klebsiella pneumoniae***, ***Acinetobacter baumannii***, ***Pseudomonas aeruginosa,*** and ***Enterobacter*** spp.) are a group of opportunistic pathogens which account for most hospital-acquired infections, and their antibiotic resistance is rising^4,5^. Rapid diagnosis and treatment of wound infection is therefore of utmost importance. Current diagnosis involves visual inspection of the wound for signs of infection (inflammation), followed by collection of swabs or tissues, microbial culture and identification in a clinical laboratory, which can take hours or even days.

Mass spectrometry (MS) has emerged as a powerful technique for identification and classification of microorganisms. Matrix-assisted laser desorption/ionization time-of-flight (MALDI-TOF) MS has been in development for that purpose since the late 1990s^6,7^ and is now well-established in the clinic^8^. In the analysis, patient-derived samples are cultured and individual colonies are smeared onto a MALDI target plate. A matrix is applied and the sample is subjected to MALDI-TOF MS. Identification is achieved by spectral fingerprinting, that is, matching protein mass spectra acquired from unknown samples against databases filled with mass spectra of previously identified microorganisms. An often-quoted advantage of MALDI-TOF is its speed. It is certainly true that the analysis time is short, especially in comparison with biochemical techniques, but this belies the time taken for culturing of the sample on solid media. Recent research has focused on the use of blood cultures to reduce the time of the sample preparation, but even with this approach it takes approximately 2 days to obtain the final result^9^. Other disadvantages of MALDI-TOF MS, particularly for the analysis of biofilms, arise because analysis takes place under vacuum. Problems include poor matrix saturation and sample flaking^10^.

The ideal solution would involve a point-of-care diagnostic in which the microorganisms are identified directly from the wound, thus reducing the time from patient to result and enabling rapid deployment of the appropriate narrow spectrum antibiotic. Previous work in our laboratory has focused on the development of liquid extraction surface analysis (LESA) MS for direct analysis of bacteria growing on solid media^11–13^. LESA MS^14^ is an ambient mass spectrometry technique, i.e., is conducted under atmospheric conditions in the open laboratory. Other ambient mass spectrometry techniques that have been applied to the analysis of bacteria include desorption electrospray ionisation (DESI) MS^15–23^, rapid evaporative ionisation mass spectrometry (REIMS)^24–27^, paper spray (PS) MS^28–30^, nano-DESI^31–33^, and Flowprobe^34^. These techniques have so far been limited to small molecules (lipids and metabolites), whereas crucially LESA MS is capable of analysing intact proteins in bacteria. The unique features of LESA MS with respect to its potential as a point-of-care diagnostic for wound infection are: (1) LESA sampling can be applied to any surface. To date, LESA sampling has been performed on substrates placed within the sampling platform; however, there is no inherent restriction on the nature of the substrate, e.g., a wound on a patient. (2) LESA MS enables the characterisation of proteins (like MALDI-TOF MS) and can be performed on living bacteria (unlike MALDI-TOF MS); (3) LESA MS has the potential to identify not just microbial proteins but also proteins from the patient, thus providing an indication of host response.

To confirm the potential of LESA MS for direct identification of bacterial wound infections, we have applied LESA MS to the analysis of bacteria growing in wounded three-dimensional in vitro living skin equivalents (“Labskin”). Labskin comprises a dermal layer, consisting of primary fibroblasts embedded in a fibrin matrix, and an epidermal layer created by seeding keratinocytes on the dermis, and the ability to culture microbes on the model is proven^35^. Clench and co-workers have applied MALDI MS and MALDI MS imaging of transverse sections of Labskin for the analysis of lipids and small molecules^36–41^. Here, we demonstrate the analysis and identification of three ESKAPE pathogens, *S. aureus*, *K. pneumoniae,* and *P. aeruginosa* directly from wounded and infected Labskin by LESA MS. For *S. aureus*, two strains were considered, an MRSA reference strain and an MSSA clinical isolate. Both bacterial and human proteins were detected and identified. Protein identification was achieved by top-down mass spectrometry^42^ in which intact protein ions are fragmented to provide sequence information. The mass-to-charge ratios of the resulting sequence fragments are searched against protein databases by use of dedicated algorithms and putative identifications and associated scores are returned. Seven human proteins and nine bacterial proteins were identified in total. Five of the identified human skin proteins are known to have antimicrobial activity. The protein δ-hemolysin was identified from both strains of *S. aureus*; however, the sequence of δ-hemolysin differs between the two, with an associated mass difference which is easily detected by mass spectrometry. Detection of these proteins therefore allows the differentiation of these species.

## Results and discussion

### Infection of the Labskin models and LESA MS analysis of Labskin samples

**Figure 1:**
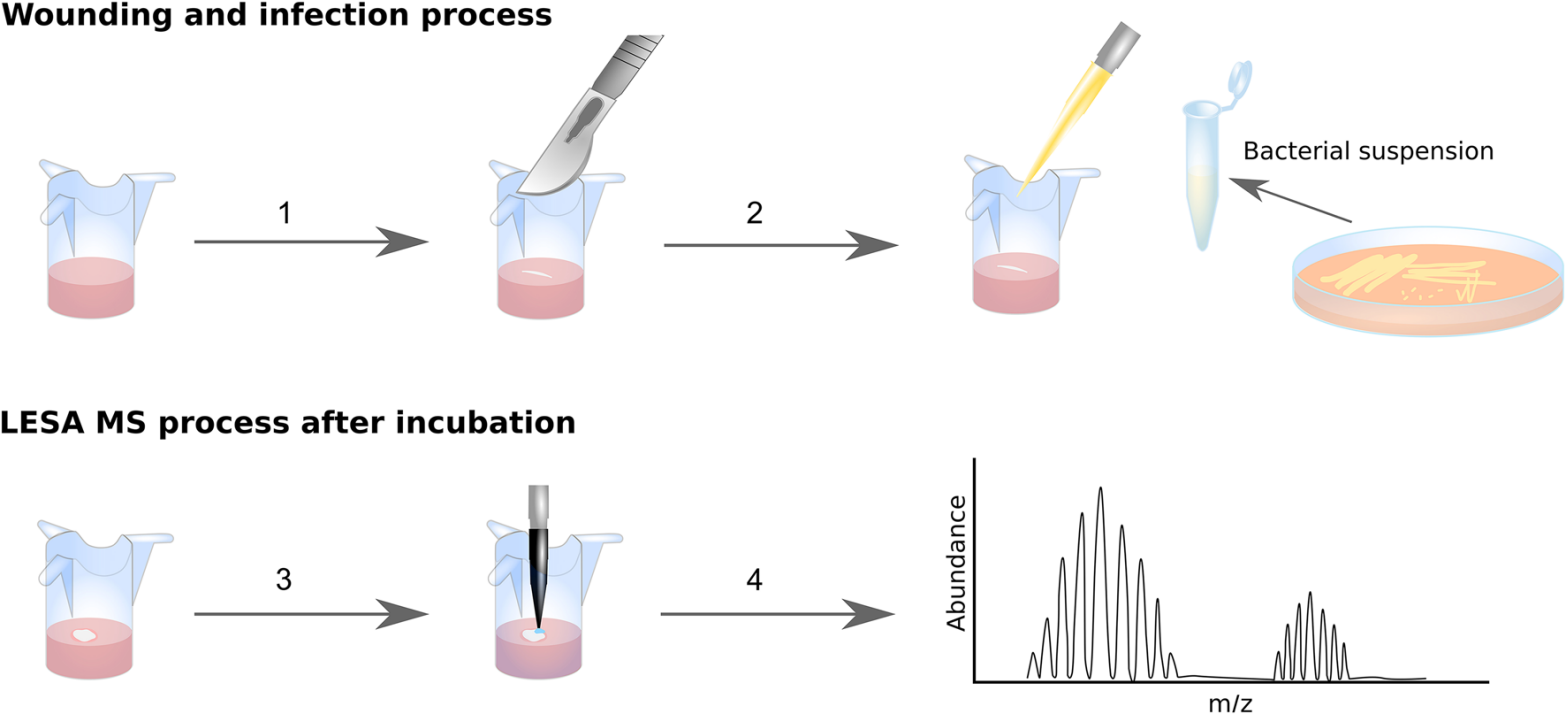
Labskin wounding, inoculation and analysis workflow. The Labskin sample (in the cell insert) is wounded with a scalpel blade (1) and inoculated with bacteria (2). After incubation (3), the infected Labskin sample is analysed by LESA MS (4).

The workflow is summarised in Fig. 1. Briefly, the Labskin samples were wounded with a scalpel blade and inoculated with bacterial suspensions of *S. aureus* NCTC13435, *S. aureus* MSSA476, *K. pneumoniae* KP257, and *P. aeruginosa* PS1054 (SI, Table S1). The sampling solvent was optimised for extraction of intact proteins from control Labskin models and determined to be ethanol, water and formic acid (60:35:5) (SI, Fig. S1). An important consideration in translation of this approach to a point-of-care diagnostic is patient-friendly extraction such as that presented by an ethanol-based solvent system. The optimised sampling solvent was subsequently validated for bacterial protein detection in LESA MS of microbial colonies growing on agar (SI, Fig. S2).

Four incubation time points were investigated: 24, 48, 72, and 96 h. After 24 h of incubation, there were no visible signs of colony growth inside the wound. The LESA mass spectra did not contain any peaks corresponding to bacterial proteins; however, several peaks corresponding to human skin proteins were observed (see below for details of protein identification).

**Figure 2:**
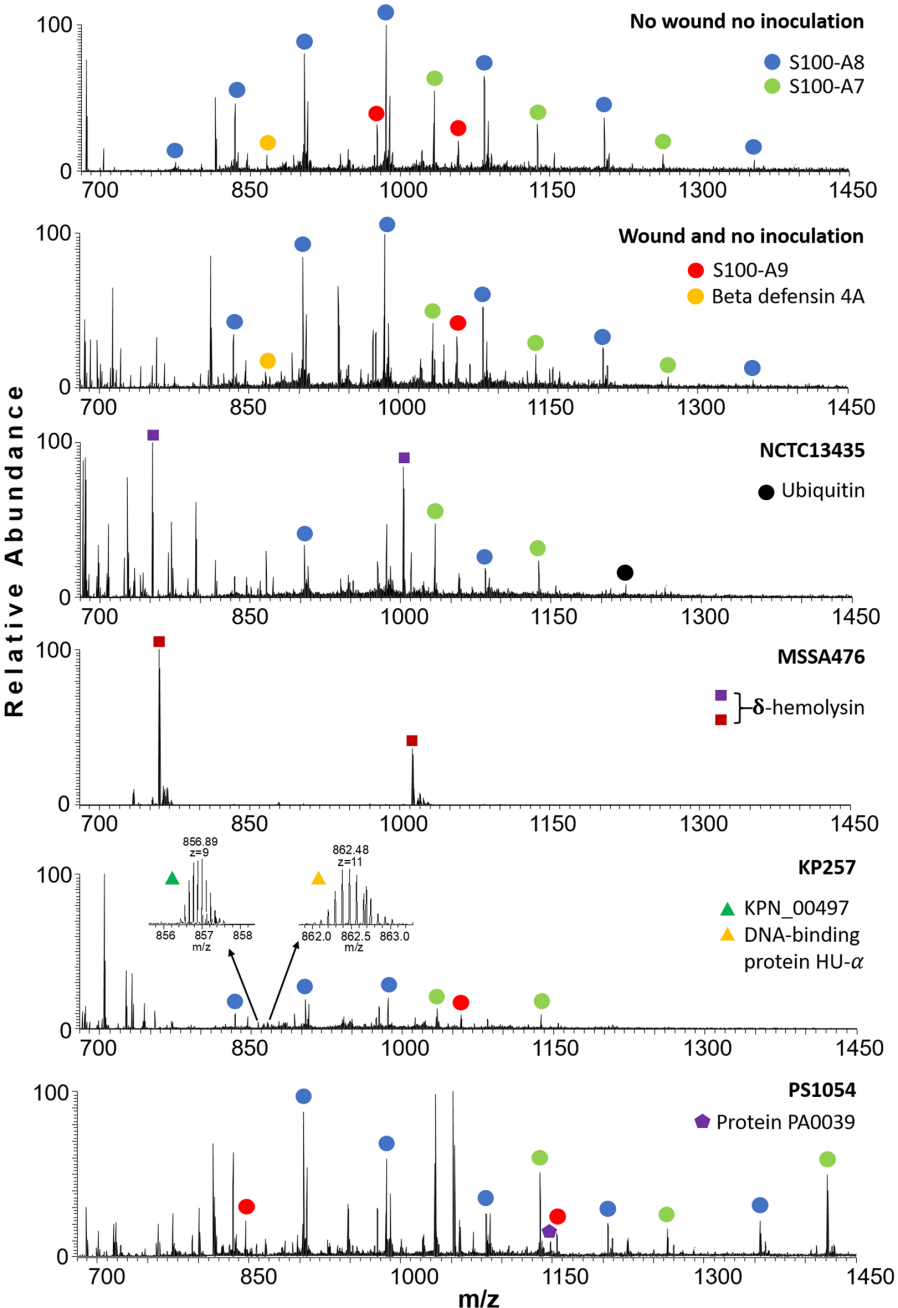
LESA mass spectra obtained from the intact control, wounded control, and Labskin samples wounded and infected with *S. aureus* NCTC13435, *S. aureus* MSSA476, *K. pneumoniae* KP257 and *P. aeruginosa* PS1054 after 48 h of incubation. Identified proteins are labelled. No bacterial proteins were detected in the control samples.

After 48 h of incubation, colonies had formed in the wound for NCTC13435, MSSA476, and KP257 (Fig. S3). For PS1054, significant changes in the Labskin structure were observed, together with the presence of the typical subtle green colour associated with secretion of pyoverdine and pyocyanin in *P. aeruginosa*^43^. Pyocyanin has molecular weight of 210.231 Da which falls below the *m*/*z* range of this experiment. The molecular weight of pyoverdine (1,365.424 Da) is within the *m*/*z* range of the experiment; however, no corresponding peaks were detected. Peaks corresponding to bacterial proteins were present in the mass spectra for all of the infected and wounded Labskin samples, in addition to peaks corresponding to human skin proteins (Fig. 2). Non-wounded Labskin samples inoculated with *S. aureus*, *K. pneumoniae,* and *P. aeruginosa* did not exhibit colony growth or changes in their structure during the experiment (Fig. S3). This result is unsurprising as colonisation of intact skin by opportunistic pathogens does not result in an infection. Comparison of the LESA mass spectra obtained from the control samples (both intact and wounded) with the infected samples (Fig. 2) reveals that no peaks corresponding to bacterial proteins were identified in the samples that were not inoculated, confirming that no bacterial cross-contamination occurred.

After 72 h incubation, two further proteins were detected in the mass spectra of the wounded samples infected with PS1054 (Fig. S4), suggesting further bacterial growth in the wound. In some LESA mass spectra obtained from the samples infected with PS1054, no skin proteins were detected, likely due to the rapid progress of the bacterial colonisation in the infected wound. After 96 h, no additional proteins, either human or bacterial, were observed.

### Bacterial and human skin proteins identified following LESA MS analysis

The human and bacterial proteins identified following LESA MS are summarized in Table 1 (details of protein assignments are given in the SI, Fig. S6–S22 and Tables S2–S18). Five of the human proteins identified (β-defensin 4A, elafin, S100A7, S100A8, and S100A9) are antimicrobial peptides (AMPs) and four (S100A6, S100A7, S100A8, and S100A9) belong to the low molecular weight S100 family of calcium-binding proteins.

**Table 1:** Summary of human skin and bacterial proteins identified from Labskin samples.

Observed monoisotopic mass (Da)	Theoretical monoisotopic mass (Da)	Mass difference (ppm)	Protein name	Uniprot accession number	Modification	Sequence coverage (%)	Sample type (*I* inoculated, *C* control)
Human skin proteins							
4,325.1296	4,325.1371	− 1.7387	β-defensin 4A	O15263	-Signal peptide (1–23), disulfide bonds (31–60, 38–53, 43–61)	12	I/C
5,994.8239	5,994.8202	0.6205	Elafin	P19957	Disulfide bonds (76–105, 83–109, 92–104, 98–113)	9	I
8,559.6029	8,559.6167	− 1.6146	Ubiquitin	P62987	–	16	I/C
10,027.3088	10,027.2911	1.7632	S100-A6	P06703	-Met; acetylation of N-terminus (A)	8	I/C
10,827.6373	10,827.6492	− 1.1046	S100-A8	P05109	–	53	I/C
11,360.4787	11,360.5189	− 3.5403	S100-A7	P31151	-Met; acetylation of N-terminus (S), E27D substitution	26	I/C
12,682.2879	12,681.2806	− 0.4274	S100-A9	P06702	Acetylation of N-terminus (S) (truncated form) S-nitrosylation (C3) (full length form))	17	I/C

Observed monoisotopic mass (Da)	Theoretical monoisotopic mass (Da)	Mass difference (ppm)	Protein name	Uniprot accession number	Modification	Sequence coverage (%)	Hours of incubation
*S. aureus* NCTC13435							
2,633.4082	2,633.4080	0.0573	Phenol-soluble modulin α3	P0C805	fMet	95	48
3,004.6109	3,004.6302	− 0.1664	δ-hemolysin	Q2FWM8	fMet	60	48
*S. aureus* MSSA476							
2,633.4094	2,633.4080	0.5240	Phenol-soluble modulin α3	P0C805	fMet	95	48
3,034.6413	3,034.6413	− 0.0066	δ-Hemolysin	Q6G7S2	fMet, G10S substitution	88	48
*K. pneumoniae* KP257							
7,698.9926	7,698.9638	3.7382	KPN_00497	A6T5S6	-Signal peptide (1–19), R49K substitution	19	48
9,471.1664	9,471.1468	2.0673	DNA-binding protein HU-α	A6TGQ7	–	30	48
*P. aeruginosa* PS1054							
5,731.9826	5,731.9816	0.1814	PA0039	Q9I793	-Signal peptide (1–21)	13	48
8,557.5098	8,557.5077	0.2431	PA4739	Q9HV60	-Signal peptide (1–32)	44	72
9,081.0514	9,081.0445	0.7576	DNA-binding protein HU-β	P05384	–	26	72

S100 proteins are present in the human body in both intracellular and extracellular forms. In its native state, S100A6 (also known as calcyclin) exists as a homodimer binding two Ca^2+^ ions. The protein was detected as a monomer in our experiments as a result of the denaturing extraction solvent system. S100A6 is known to be overexpressed in skin melanomas^44^. It is also known that S100A6 is upregulated by epidermal growth factor (EGF) and foetal calf serum^45,46^, both of which are used in the construction of the skin model^35^ and may explain the presence of S100A6 in the mass spectra. S100A7 (also known as psoriasin) is a calcium and zinc-binding protein expressed by normal cultured (and malignant) keratinocytes^47^, and therefore the presence on the skin models might be expected. Studies have shown that this protein has antimicrobial activity against *Escherichia coli*, whilst also targeting *S. aureus*, *S. epidermidis,* and *P. aeruginosa,* albeit less effectively^48^. S100A8 (calgranulin A) and S100A9 (calgranulin B) typically exist as the heterodimer calprotectin, which exhibits antimicrobial activity, but both are also known to act separately^49,50^. Both proteins were detected (as monomers) in the mass spectra. S100A9 was identified in two different forms. One form was truncated at the N-terminus and the sequence was identified with serine acetylation at the (new) N-terminus. The second form was the full length protein, with S-nitrosylated cysteine at position 3, as indicated by UniProt. Elevated expression of calprotectin may be induced by skin dehydration, i.e., transepithelial water loss (TEWL)^51^. TEWL is known to be greater for skin models when compared to real skin^35^, and this may explain the high abundance of S100A8 and S100A9 in the mass spectra. The function of S100A8/A9 is also believed to be important in wound healing tissue^52^, however S100A8 was detected in all of the Labskin samples, including non-wounded controls.

Proteins identified outside of the S100 family included β-defensin 4A, elafin, and ubiquitin. Antimicrobial β-defensin 4A (or β-defensin 2) protects the skin from gram-positive and gram-negative bacteria^53^. This protein contains three disulphide bonds, specific to the family of defensins^54^. The high number of disulphide bonds and relatively short amino acid sequence resulted in poor sequence coverage (7.5%) but with a high confidence in the manual fragment assignment (see SI, Fig. S6, Table S2). Peaks corresponding to β-defensin 4A were detected in all mass spectra regardless of the presence of wound or infection. The expression of β-defensin 4A has been shown to be upregulated in wounded and infected skin^55^. In our experiment, the abundance of β-defensin 4A ions was higher in the control samples (both intact and wounded) when compared to the infected and wounded skin models. The higher abundance suggests that this protein may be a potential biomarker for infected tissue; however, validation experiments would be required. Elafin (skin antileucoprotease—SKALP, elastase specific inhibitor) is a peptide whose functions include antimicrobial activity and inhibition of proteases^56^. Its expression has been shown to be upregulated during inflammation, and it is a biomarker for graft-versus-host disease^57^. In our experiments, elafin was only detected in the mass spectra obtained from non-wounded skin samples which had been inoculated with *P. aeruginosa*. Finally, ubiquitin was observed in the majority of the acquired mass spectra. Ubiquitin is also commonly observed in the LESA mass spectra of tissue sections^58^.

In terms of bacterial proteins, both extracellular and intracellular proteins were identified, in agreement with our previous findings for *S. aureus* MSSA476 and *P. aeruginosa* PS1054 grown on agar^12^. For both *S. aureus* strains, peaks were detected which correspond to two extracellular toxins – phenol-soluble modulin α3 and δ-hemolysin. These highly cytolytic peptides belong to the group of phenol-soluble modulins specific to *S. aureus* species^59^. Top-down LESA MS revealed that both had formylated methionine at their N-termini. The accumulation of formylated δ-hemolysin is known to occur during the post-exponential growth phase^60^, reflecting our findings that the abundance of this protein increases with incubation time.

In the wounded skin samples infected with *K. pneumoniae* KP257, two proteins were identified. Firstly, an uncharacterized protein predicted on the basis of gene KPN_00497 was identified. The protein was detected with a cleaved signal peptide (1–19) and a R → K substitution at position 49. Secondly, DNA-binding protein HU-α, which is involved in stabilization of DNA under extreme environmental conditions, was identified.

Three bacterial proteins were identified from the samples infected with *P. aeruginosa* PS1054: two uncharacterized proteins and DNA-binding protein HU-β. All have been identified previously from colonies of PS1054 growing on agar^12^. The first uncharacterized protein is predicted on the basis of gene PA0039 with signal peptide (1–21) cleaved. PA0039 was the only protein detected in the mass spectra after 48 h that was sufficiently abundant to perform top-down tandem mass spectrometry (MS/MS) analysis. The second uncharacterized protein is predicted on the basis of gene PA4739. Information about this protein available from UniProt suggests that the signal peptide is cleaved after amino acid 25, however our results confirm that the signal peptide cleaves after amino acid 32, as observed previously^12^. PA4739 and DNA-binding protein HU-β were both detected after 72 h of incubation.

### Mass difference between the δ-hemolysins of *S. aureus* strains

The sequence of δ-hemolysin differs between the two *S. aureus* strains NCTC13435 and MSSA476 as a result of a glycine to serine substitution at position 10 (G10S). This is an allelic variant related to a mutation in the hld gene and is characteristic of certain ST1 and ST59 strains of *S. aureus*, including ST1 strain MSSA476^61^. The G → S substitution results in a mass difference, Δm =  + 30.0105 Da, which can be used to differentiate between the two strains by LESA MS. (The site of substitution can be confirmed by LESA tandem mass spectrometry (MS/MS), see Fig. S5, SI). That is, LESA MS presents a rapid tool for direct differentiation between allelic variants in strains of *S. aureus*.

## Conclusion

We have demonstrated the rapid identification of ESKAPE pathogens, including different strains of *S. aureus*, directly from infected wounds in 3D in vitro living skin equivalents. Our approach involves LESA mass spectrometry, an ambient technique which here makes use of ethanol-based solvents. Intact proteins from both human skin and the infecting bacteria were identified with high confidence. Bacterial proteins could be identified within minutes once visible signs of infection were apparent (i.e., 48 h after inoculation of the skin model with bacteria). Detection of allelic variants of the protein δ-hemolysin enabled differentiation between the two strains of *S. aureus.* Future work will focus on identification of proteins from mixtures of pathogens in infected wounds. Furthermore, improvements in mass spectrometry technology will likely be accompanied by improved numbers of proteins detected from the various microbial species.

## Materials and methods

### Materials

Analytical grade acetonitrile, ethanol, water and formic acid were purchased from Fisher Scientific (Loughborough, UK). LB broth [yeast extract (VWR, Lutterworth, UK), peptone (Sigma Aldrich, Gillingham, UK) and sodium chloride (Fisher Scientific, Loughborough, UK)] and LB agar [LB broth with added agar (Appleton Woods, Birmingham, UK)] were used for bacterial culture. In vitro 3D skin models “Labskin” were purchased from Innovenn (Sand Hutton, UK). *S. aureus* NCTC13435 was obtained from Public Health England (Porton Down, UK) via Innovenn. *S. aureus* MSSA476, *P. aeruginosa* PS1054 were obtained from Mark Webber (Institute of Microbiology and Infection, University of Birmingham, UK) and *K. pneumoniae* KP257 was obtained from Willem van Schaik (Institute of Microbiology and Infection, University of Birmingham, UK).

### Skin sample preparation

On arrival, Labskin samples were transferred into a new 12-well plate with fresh proprietary Labskin medium (Innovenn, Sand Hutton, UK) and incubated overnight at 37 °C, 5% CO_2_ and > 95% relative humidity. On day 2, the medium was replaced with the fresh Labskin medium, and the samples were wounded with a scalpel blade (Fig. 1). The wounded samples were infected with *S. aureus* NCTC13435 (n = 9), *S. aureus* MSSA476 (n = 3), *K. pneumoniae* KP257 (n = 3) and *P. aeruginosa* PS1054 (n = 8). Full details of the infection process and calculation of infectious dose is given in *Supplementary Information*. Three control samples were included: intact (n = 5), wounded but not inoculated (n = 5) and inoculated but not wounded for *S. aureus* NCTC13435 (n = 4), *S. aureus* MSSA476 (n = 2), *K. pneumoniae* KP257 (n = 2) and *P. aeruginosa* PS1054 (n = 7). All samples (control and infected/wounded) were incubated for 24, 48, 72 and 96 h after the infection at 37 °C, 5% CO_2_ and > 95% relative humidity prior to MS analysis.

### LESA mass spectrometry

LESA MS was performed using an Advion Triversa Nanomate (Advion, Ithaca, NY, USA) coupled to a Thermo Orbitrap Elite mass spectrometer or a Q Exactive HF mass spectrometer (both Thermo Fisher Scientific, Bremen, Germany) as described previously^11,12^. The solvent system used for LESA MS comprised ethanol (Fisher Scientific, Loughborough, UK), water (Fisher Scientific, Loughborough, UK) and formic acid (Fisher Scientific, Loughborough, UK) (60:35:5). Labskin samples were placed in the 60 mm Petri dishes adjacent to the half of the 96-well microtiter plate. The robotic arm of the Triversa Nanomate aspirated 3 μL of the extraction solvent system from the microtiter plate. Next, the robotic arm was relocated to a position above the sample, descended above the Labskin surface such that the formation of the liquid microjunction was allowed and dispensed 2 μL of the solvent system. After the sampling process, 2.5 μL of the solvent system with extracted analytes was re-aspirated back into the pipette tip and introduced into the mass spectrometer via chip-based nano-electrospray system at gas pressure 0.3 psi and a tip voltage 1.75 kV. The Triversa Nanomate was controlled with the advanced user interface (AUI) in the Chipsoft software 8.3.1. (Advion, Ithaca, NY, USA). The mass spectra were recorded for at least 3 min in positive ion mode in full scan mode, 600–2000 *m*/*z,* at resolution 120,000 at 400 *m*/*z*. Top-down MS/MS analysis of bacterial proteins was carried out using both collision induced dissociation (CID) in the ion trap using helium gas at a normalized collision energy 35% (Orbitrap Elite) and higher energy collision dissociation (HCD) at a varying collision energy between 15–60 eV depending on the protein charge state (Q Exactive HF). When performing fragmentation, each scan comprised 30 co-added microscans (Orbitrap Elite) or 5 co-added microscans (Q Exactive HF) and data were recorded for at least 5 min.

### Data processing and protein identification

Top-down identification of proteins was performed by use of ProSight 4.1 software (Thermo Fisher Scientific, Bremen, Germany) as described previously^12^. Pre-built ProSight top-down protein databases for each model organism were downloaded from the Database Warehouse of Proteinaceous website (https://www.proteinaceous.net), which also includes link to Uniprot proteomes with the Proteome ID listed. Databases included *Homo sapiens* (UP000005640, 71,599 entries), *Staphylococcus aureus* NCTC 8325 (UP000008816, 2,889 entries), *Klebsiella pneumoniae* ATCC 700721 (UP000000265, 5,126 entries), and *Pseudomonas aeruginosa* ATCC 15692/PA01 (UP000002438, 5,563 entries). MS/MS spectra were imported into ProSightPC in profile mode and deconvoluted by the THRASH algorithm at default settings and S/N 3. Search parameters in absolute mass search mode accounted for all post-translational modifications and delta-mass (Δm) mode on for locating the unknown modifications or possible mutations. The search window width was 1,000 Da, with initial fragment mass tolerance ± 15 ppm and minimum matching fragments number set to 4. All identified protein sequences were subsequently checked with the Sequence Gazer function of ProSight software followed by manual peak assignment with the fragment tolerance narrowed to ± 5 ppm.

## Supplementary information

Supplementary Information.

## Data Availability

Supplementary data supporting this research is openly available from the University of Birmingham data archive at https://doi.org/10.25500/edata.bham.00000489.
