# Supplementary material for: Direct identification of bacterial and human proteins from infected wounds in living 3D skin models
Source: Sci Rep. 2020 Jul 17;10:11900. doi: 10.1038/s41598-020-68233-6 (PMC7368034; doi:10.1038/s41598-020-68233-6)
Supplement: Supplementary file 1 — Supplementary Information. [file 41598_2020_68233_MOESM1_ESM.docx]

**Supporting Information**

**Direct Identification of Bacterial and Human Proteins from Infected Wounds in Living 3D Skin Models**

Jana Havlikova^a,b^, Robin C. May^b,c^, Iain B. Styles^a,d^ and Helen J. Cooper^b^

^a^EPSRC Centre for Doctoral Training in Physical Sciences for Health, University of Birmingham, Edgbaston, Birmingham B15 2TT, United Kingdom

^b^School of Biosciences, University of Birmingham, Edgbaston, Birmingham B15 2TT, United Kingdom

^c^Institute of Microbiology and Infection, University of Birmingham, Edgbaston, Birmingham B15 2TT, United Kingdom

^d^School of Computer Science, University of Birmingham, Edgbaston, Birmingham B15 2TT, United Kingdom

**Corresponding Author:**

Email: [h.j.cooper@bham.ac.uk](mailto:h.j.cooper@bham.ac.uk)

This PDF file includes:

Preparation of bacterial solutions for infection process of skin samples

Optimisation of extraction solvent system for Labskin experiments

Protein IDs of all of the identified proteins

Figures S1 to S5

Table S1

**Preparation of bacterial solutions for infection process of skin samples**

The infectious dose in this experiment represents the minimum number of colony forming units (CFUs) necessary for development of infection. The required infectious dose was determined based on information provided by Labskin supplier Innovenn (*S. aureus*), literature values^1^ (*P. aeruginosa*) or inferred experimentally by inoculating Labskin samples with 3 different bacterial concentrations *(K. pneumoniae)*. The infectious doses are also in agreement with typical concentration range (1 – 100 CFU/mm^2^) of nosocomial pathogens on surfaces in hospital settings^2^. To ensure the correct infectious doses were administered, *S. aureus* NCTC13435, *S. aureus* MSSA476, *P. aeruginosa* PS1054 and *K. pneumoniae* KP257 were grown overnight on standard Lysogeny broth agar medium. Approximately one loop (10 μL) of each bacterium was resuspended in deionised sterile water and vortexed. The optical density (OD) of each solution was measured at 600 nm and, if necessary, the solution was diluted to an OD of 0.2. Solutions with OD 0.2 were further diluted to either 1:10000 or 1:100000 to obtain the required infectious dose. The diluted bacterial suspensions were re-grown on the LB agar overnight and colonies were counted. CFU/mL in the suspension was back-calculated, serving as an estimate of the infectious dose administered (see Tab. S 1).

Tab. S 1 Infectious doses for each bacterium. The ratio in brackets corresponds to dilution of the solution with OD 0.2.

| **Bacterial strain** | **Required infectious dose (CFU)** | **CFU/mL at**  **OD 0.2^a^** | **Volume used for infection [μL]** | **Back-calculated values of CFU/mL** |
| --- | --- | --- | --- | --- |
| *S. aureus* NCTC13435 | 100 | 3.4 x 10^8^ | 4 (1:13600) | 6.4 x 10^7^ |
| *S. aureus* MSSA476 | 120 | 3.0 x 10^8^ | 4 (1:10000) | 5.7 x 10^7^ |
| *K. pneumoniae* KP257 | 80 | 8.6 x 10^7^ | 10 (1:100000) | 4.4 x 10^7^ |
| *P. aeruginosa* PS1054 | 15 | 6.7 x 10^8^ | 3 (1:100000) | 3.7 x 10^8^ |

^a^As determined in previous colony counting experiments.

**Optimization of extraction solvent system for Labskin experiments**

Two extraction solvent systems were tested, an acetonitrile-based (acetonitrile:water:formic acid, 50:45:5) and ethanol-based (ethanol:water:formic acid, 60:35:5). Both solvent systems were capable of extracting both human and bacterial proteins; however, a higher S/N was observed with the ethanol-based solvent system. The ethanol-based solvent system was therefore used for further experiments.

Fig. S 1 Comparison of mass spectra when (top) acetonitrile-based solvent system and (bottom) ethanol-based solvent system were used for protein extraction. Both mass spectra were recorded for 3 min.

**Ethanol-based extraction solvent system for detection of proteins from bacterial colonies**

| **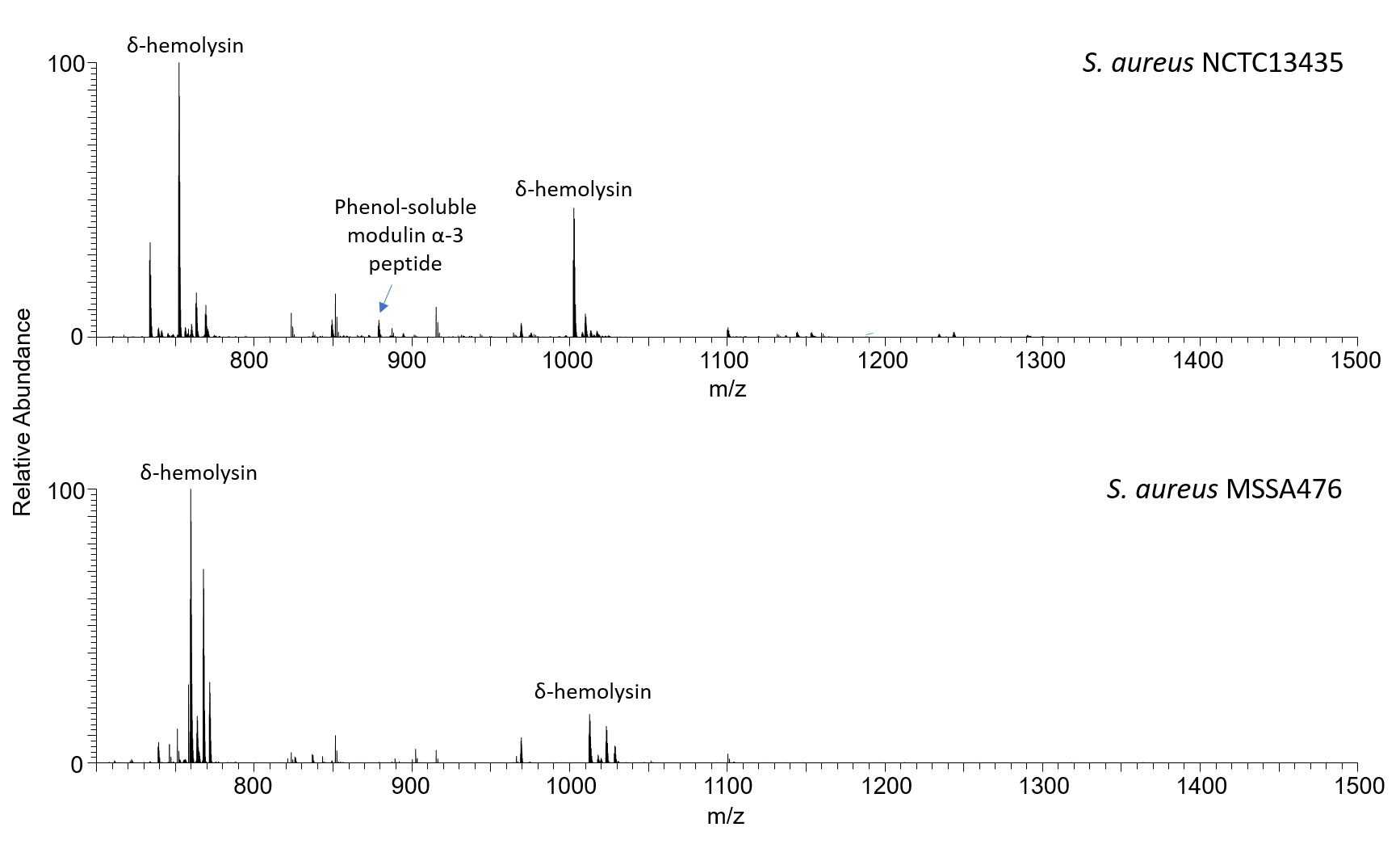** |
| --- |
| **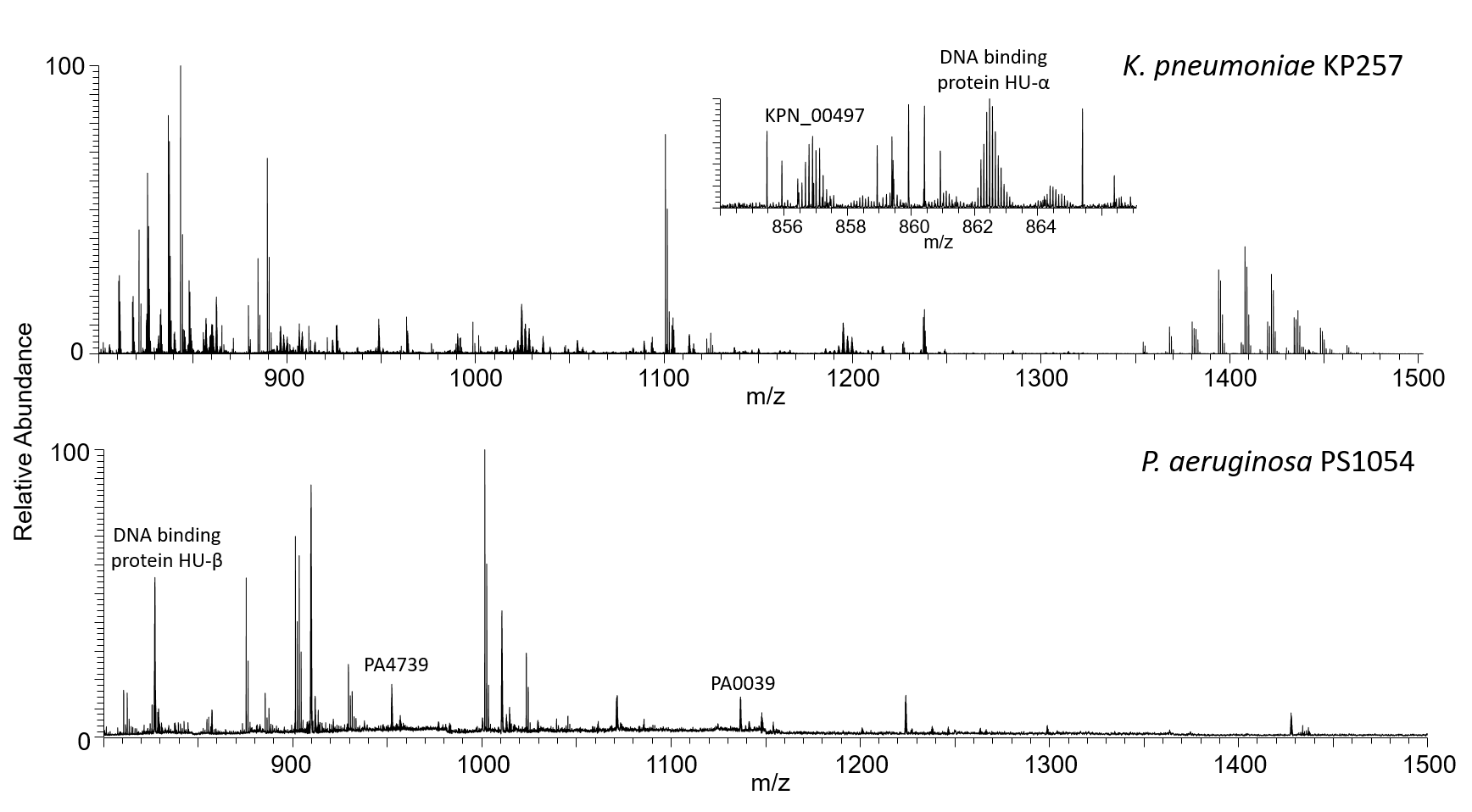** |

Fig. S 2 Example of mass spectra of bacterial colonies sampled with ethanol-based solvent (60:35:5, ethanol:water:formic acid). All of the mass spectra were acquired for 3 min. The LESA extraction was performed from bacterial colonies growing on LB agar. The sampling conditions were as described in Materials and Methods.

**Visual comparison of colonies formed inside the wounded skin model**

| 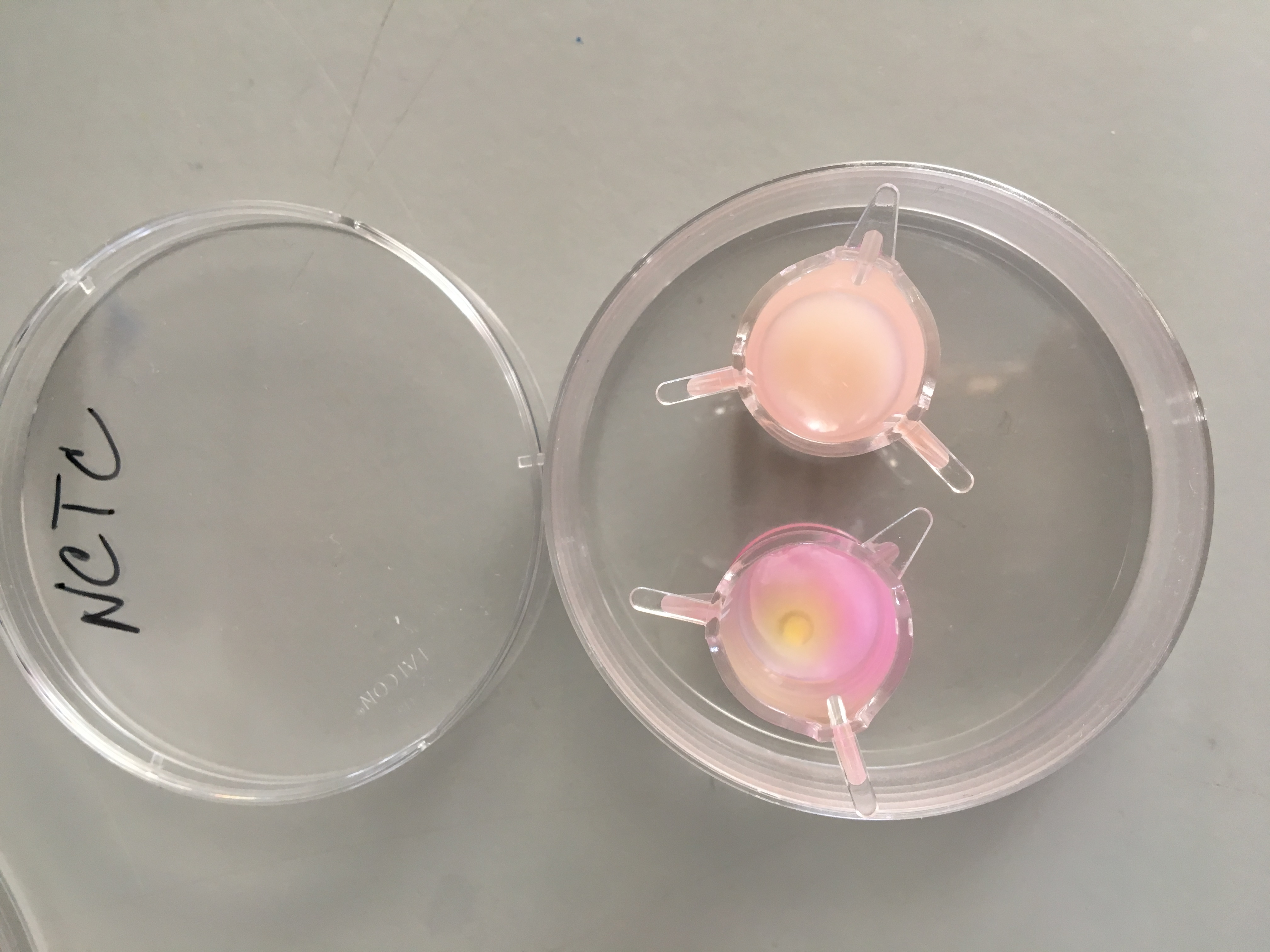  Top view  ***S. aureus* NCTC13435** | 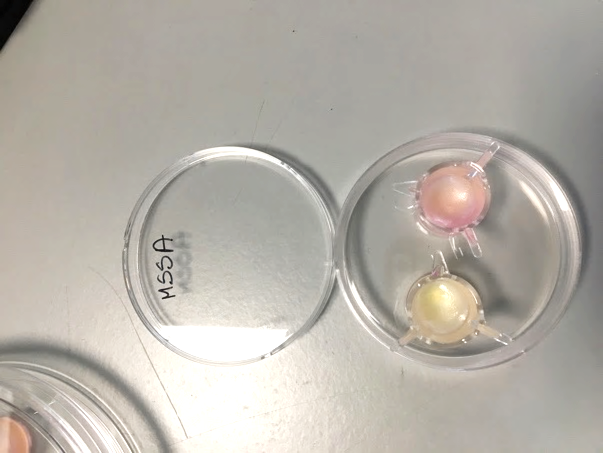  Top view  ***S. aureus* MSSA476** |
| --- | --- |
| 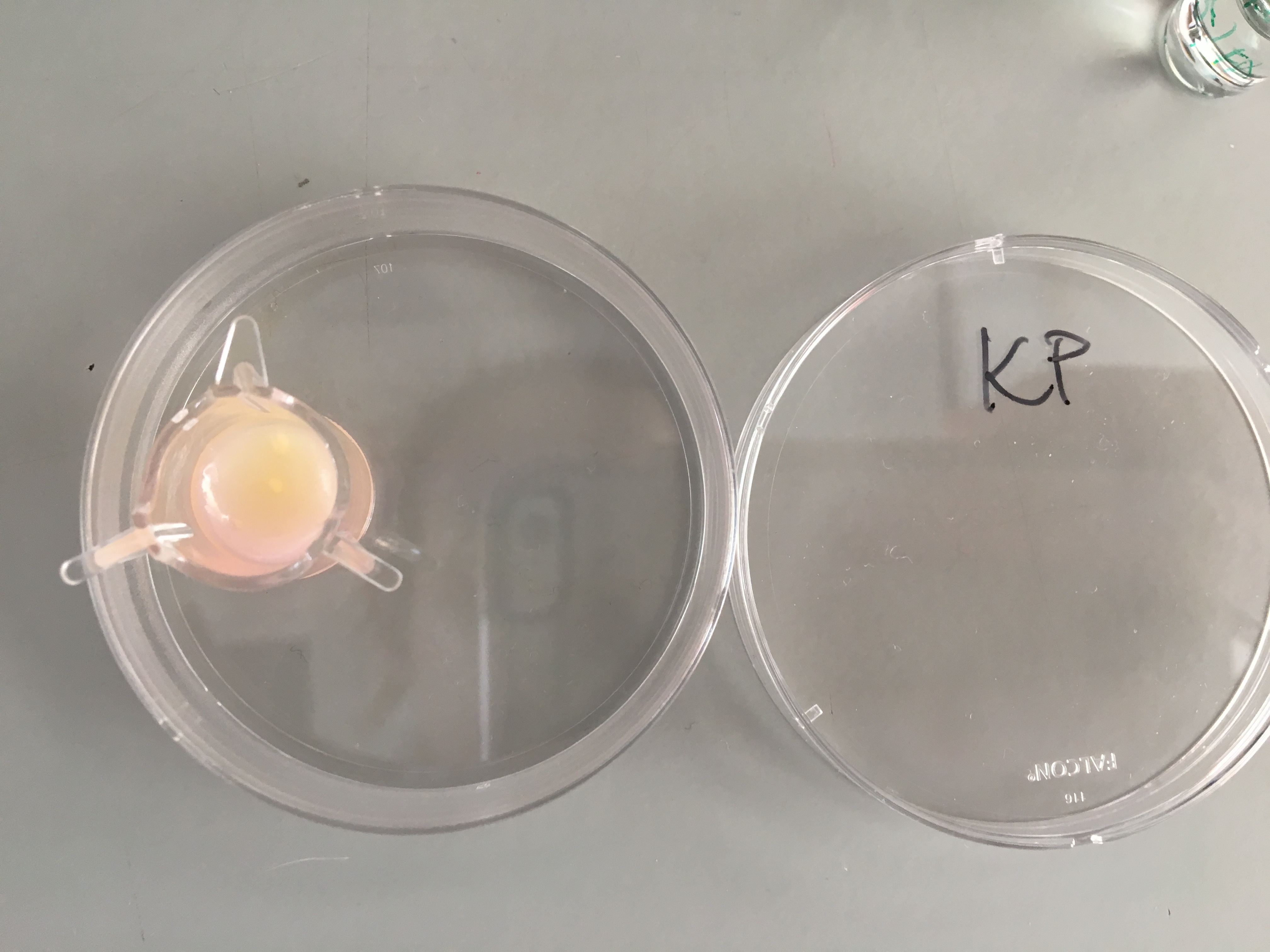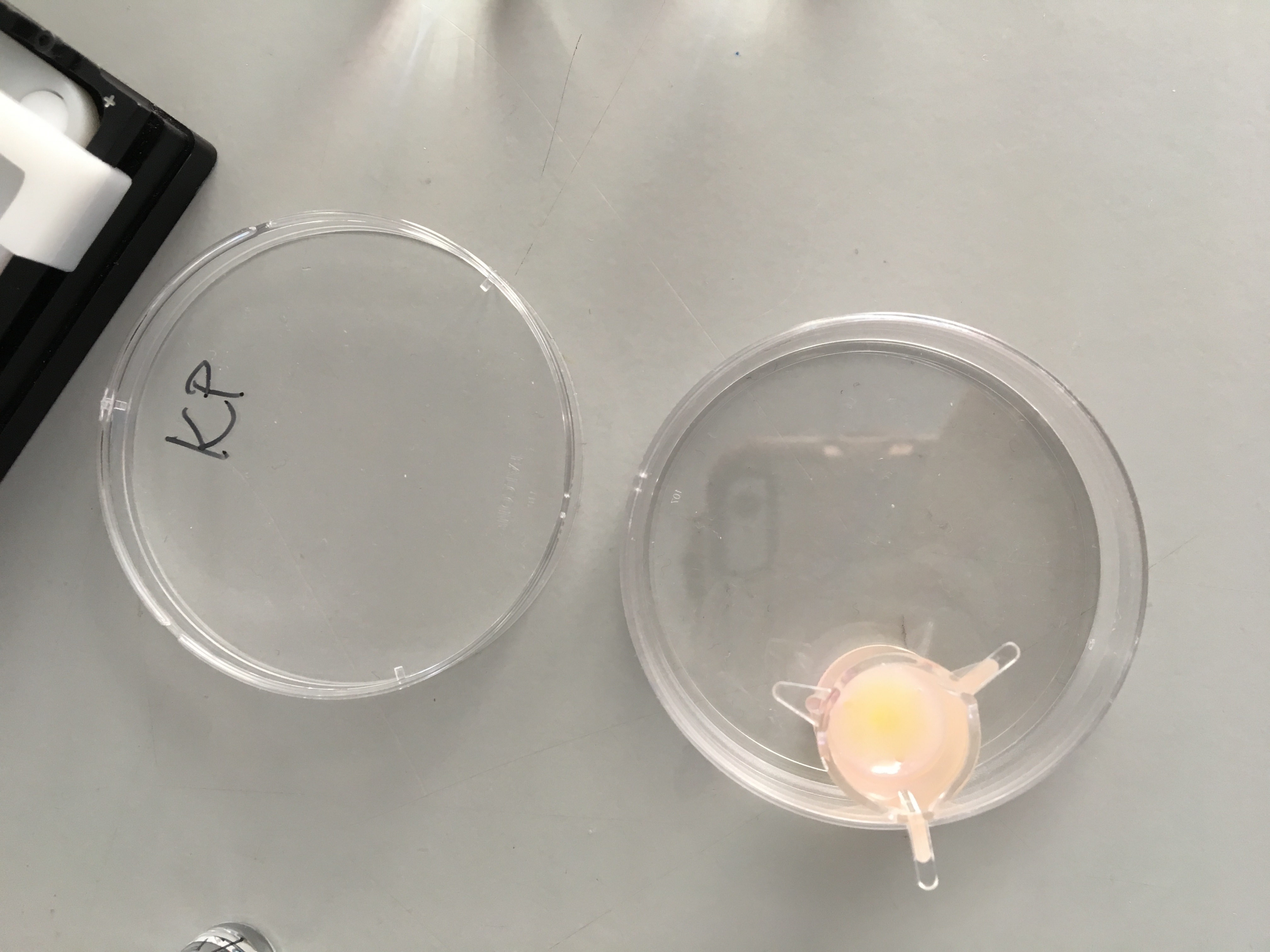  Inoculated but not wounded  Infected and wounded  Top view  ***K. pneumoniae* KP257** | |
| 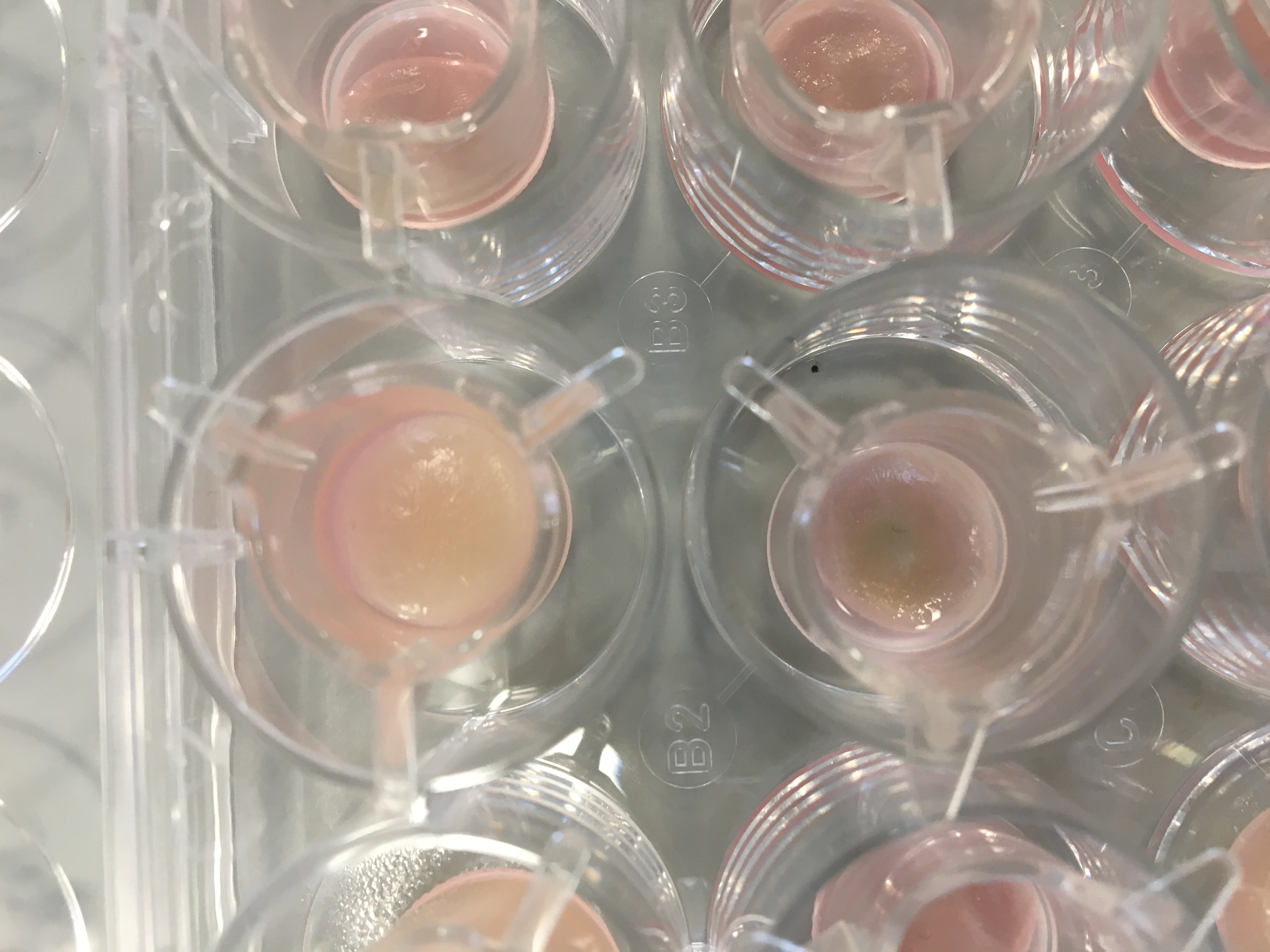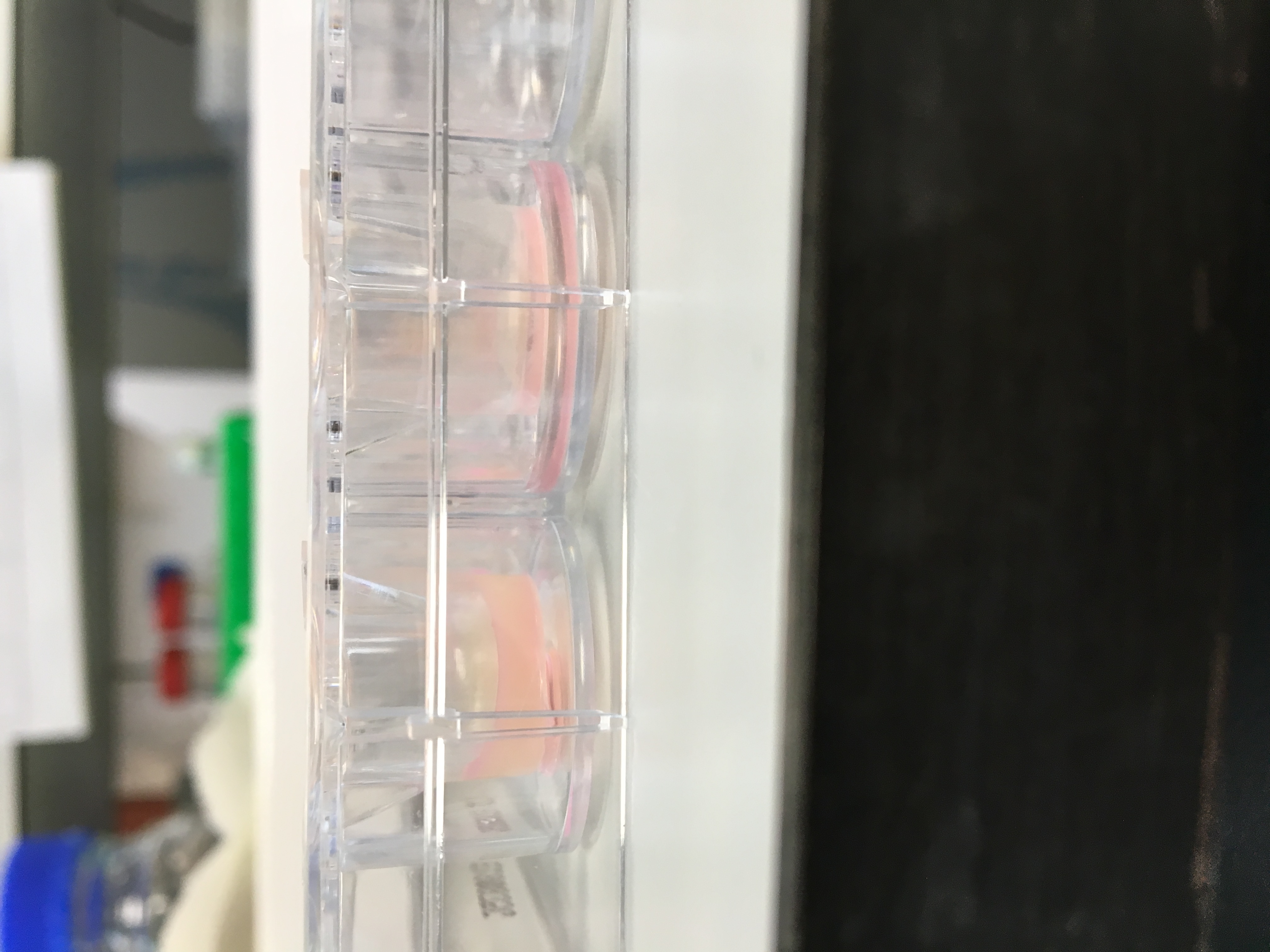  Top view  Side view  ***P. aeruginosa* PS1054** | |

Fig. S 3 Visual comparison of infected/wounded (left) and inoculated/not wounded (right) Labskin samples for each of the bacterium studied. Red circles indicate colonies formed inside the wound. In the case of P. aeruginosa, the infection development is different and leads to thinning of the Labskin model (see side view).

**Additional proteins identified from *P. aeruginosa* after 72 hours of incubation
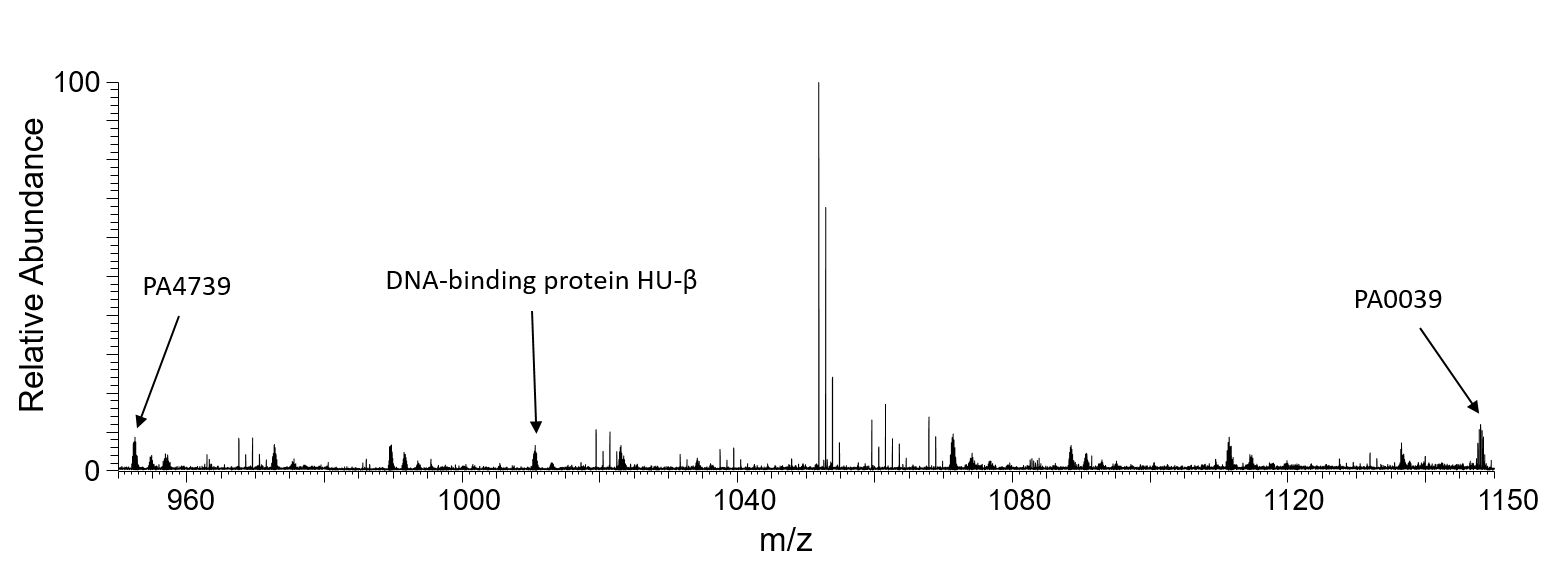
**

Fig. S 4 LESA mass spectrum obtained from P. aeruginosa 1054 after 72 hours of incubation. Proteins PA4739 and DNA-binding protein HU-β were identified.

**Mass difference between the δ-hemolysins of *S. aureus* strains**

**
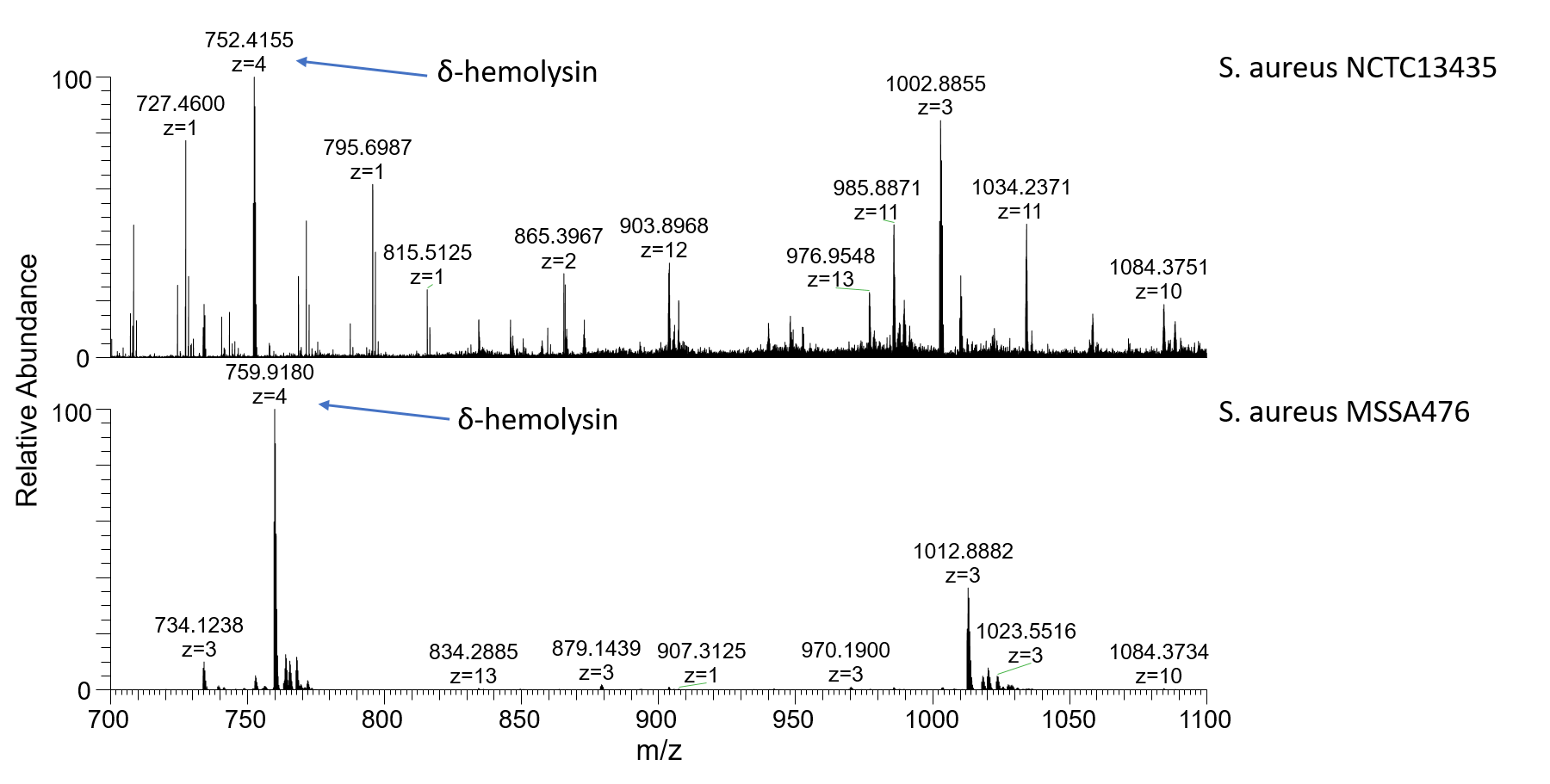
**

Fig. S 5 LESA mass spectra obtained from S. aureus NCTC13435 and S. aureus MSSA476. A mass shift of 30.01 Da is detected between the variants of δ-hemolysin from the two strains.

**Protein identification**

All masses listed are monoisotopic. Post-translational modifications: a – acetylation, f – formylation. Fragmentation methods: CID – collision induced dissociation, HCD – higher energy collision dissociation.

**Human skin proteins**

Protein name: β-defensin 4A

Charge state: 5+, CID

Sequence: GIGDPVTCLKSGAICHPVFCPRRYKQIGTCGLPGTKCCKKP (3 disulfide bonds)

**
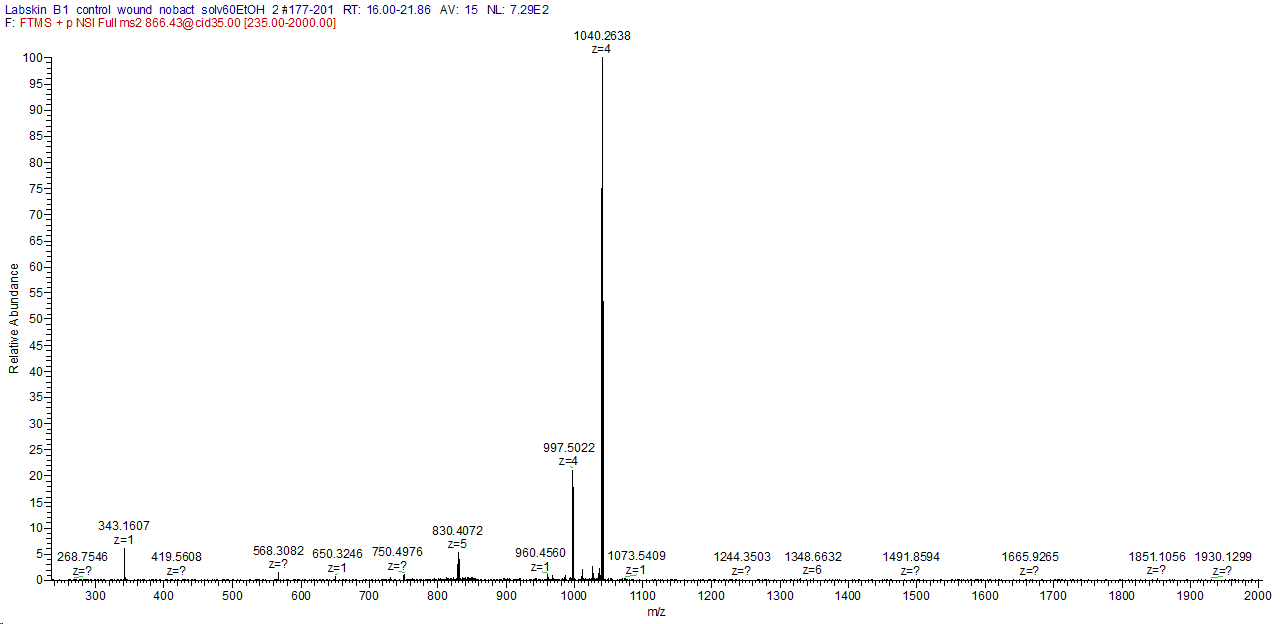
**

Fig. S 6 MS/MS mass spectrum of β-defensin 4A.

Tab. S 2 List of MS/MS fragment assignments for β-defensin 4A.

| Observed mass *m/z* | Theoretical mass *m/z* | Charge | Fragment | Mass error [Da] | Mass error [ppm] |
| --- | --- | --- | --- | --- | --- |
| 343.1607 | 343.1612 | 1 | b4 | -0.0005 | -1.457041181 |
| 996.7506 | 998.264 | 4 | y37 | -0.0068 | -1.702956332 |
| 1025.5071 | 1027.0207 | 4 | y38 | -0.0076 | -1.850011397 |
| 1039.7628 | 1041.2761 | 4 | y39 | -0.0064 | -1.53657613 |

Protein name: Elafin

Charge state: 6+, CID

Sequence: AQEPVKGPVSTKPGSCPIILIRCAMLNPPNRCLKDTDCPGIKKCCEGSCGMACFVPQ

**
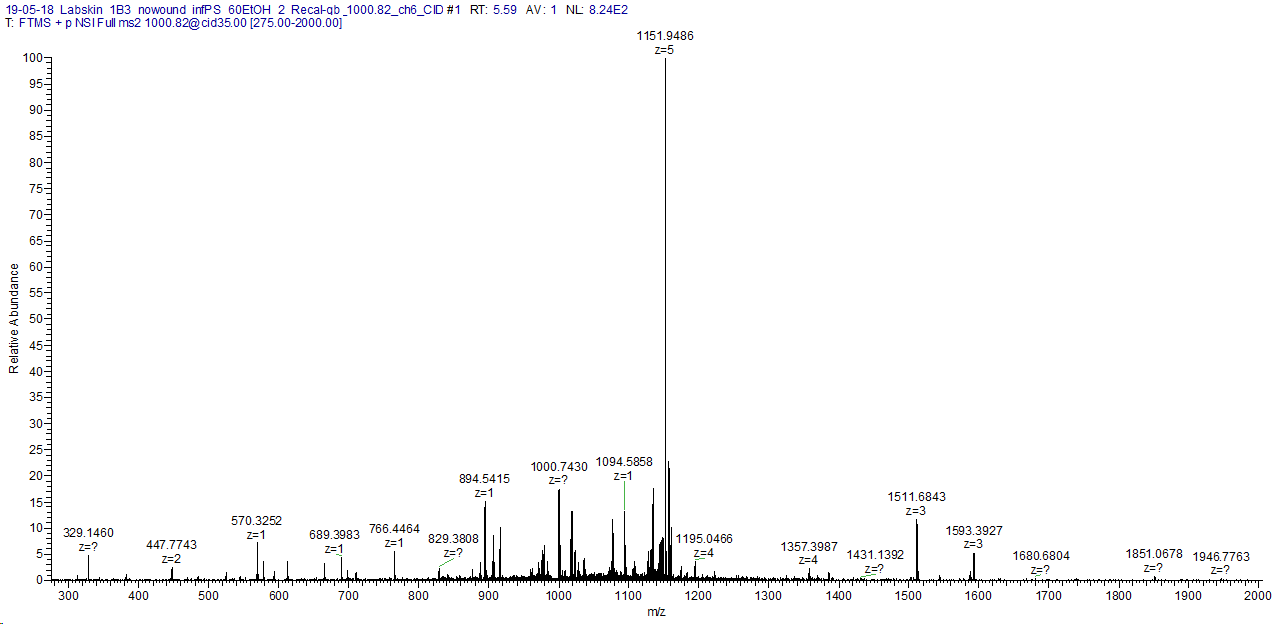
**

Fig. S 7 MS/MS mass spectrum of elafin.

Tab. S 3 List of MS/MS fragment assignments for elafin.

| Observed mass *m/z* | Theoretical mass *m/z* | Charge | Fragment | Mass difference [Da] | Mass difference [ppm] |
| --- | --- | --- | --- | --- | --- |
| 329.1459 | 329.1456 | 1 | b3 | 0.0003 | 3.0382 |
| 611.8436 | 611.8431 | 2 | b12 | 0.0010 | 0.8172 |
| 906.5047 | 906.5043 | 1 | b9 | 0.0004 | 1.1031 |
| 1151.3477 | 1151.3470 | 5 | b55 | 0.0037 | 0.1737 |
| 1160.1514 | 1160.1522 | 5 | y55 | -0.0038 | 0.1724 |

Protein name: Ubiquitin

Charge state: 7+, CID

Sequence: MQIFVKTLTGKTITLEVEPSDTIENVKAKIQDKEGIPPDQQRLIFAGKQLEDGRTLSDYNIQKESTLHLVLRLRGG


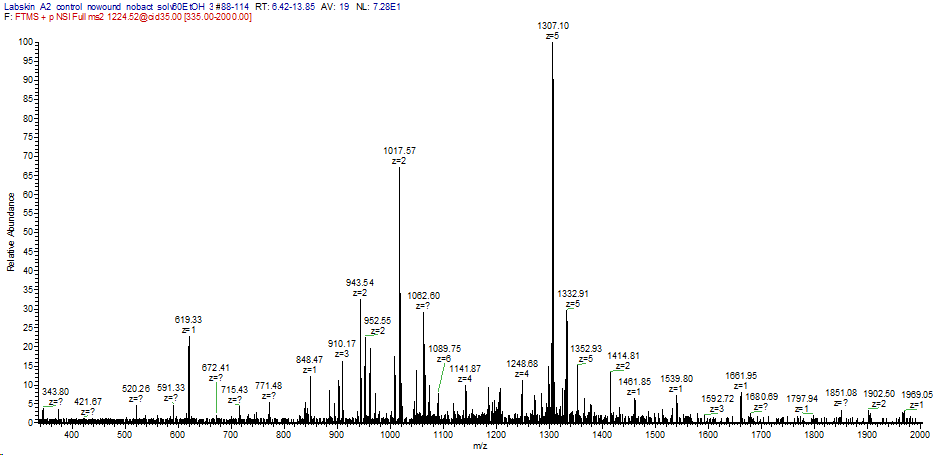


Fig. S 8 MS/MS mass spectrum of ubiquitin.

Tab. S 4 List of MS/MS fragment assignments for ubiquitin.

| Observed mass *m/z* | Theoretical mass *m/z* | Charge | Fragment | Mass error [Da] | Mass error [ppm] |
| --- | --- | --- | --- | --- | --- |
| 619.3265 | 619.3272 | 1 | b5 | -0.0007 | -1.1303 |
| 848.4687 | 848.4699 | 1 | b7 | -0.0012 | -1.4143 |
| 894.0087 | 894.0104 | 2 | b16-H2O | -0.0034 | -1.9015 |
| 903.0151 | 903.0157 | 2 | b16 | -0.0012 | -0.6644 |
| 909.5058 | 909.5069 | 3 | y24 | -0.0033 | -1.2094 |
| 943.5434 | 943.5446 | 2 | b17-H2O | -0.0024 | -1.2718 |
| 952.5491 | 952.5499 | 2 | b17 | -0.0016 | -0.8399 |
| 961.553 | 961.5539 | 1 | b8 | -0.0009 | -0.9360 |
| 1008.0652 | 1008.0659 | 2 | b18-H2O | -0.0014 | -0.6944 |
| 1017.0699 | 1017.0712 | 2 | b18 | -0.0026 | -1.2782 |
| 1049.0997 | 1049.1 | 2 | y18 | -0.0006 | -0.2860 |
| 1141.3695 | 1141.3702 | 4 | y40 | -0.0028 | -0.6133 |
| 1270.6899 | 1270.6881 | 5 | b57 | 0.009 | 1.4166 |
| 1306.5037 | 1306.505 | 5 | y58 | -0.0065 | -0.9950 |
| 1332.3128 | 1332.3136 | 5 | y59 | -0.004 | -0.6005 |
| 1352.1224 | 1352.1273 | 5 | y60 | -0.0245 | -3.6239 |
| 1436.7702 | 1436.7731 | 3 | b39 | -0.0087 | -2.0184 |
| 1461.8475 | 1461.8498 | 1 | b13 | -0.0023 | -1.5733 |

Protein name: S100-A6

Charge state: 9+, CID

Sequence: aACPLDQAIGLLVAIFHKYSGREGDKHTLSKKELKELIQKELTIGSKLQDAEIARLMEDLDRNKDQEVNFQEYVTFLGALALIYNEALK


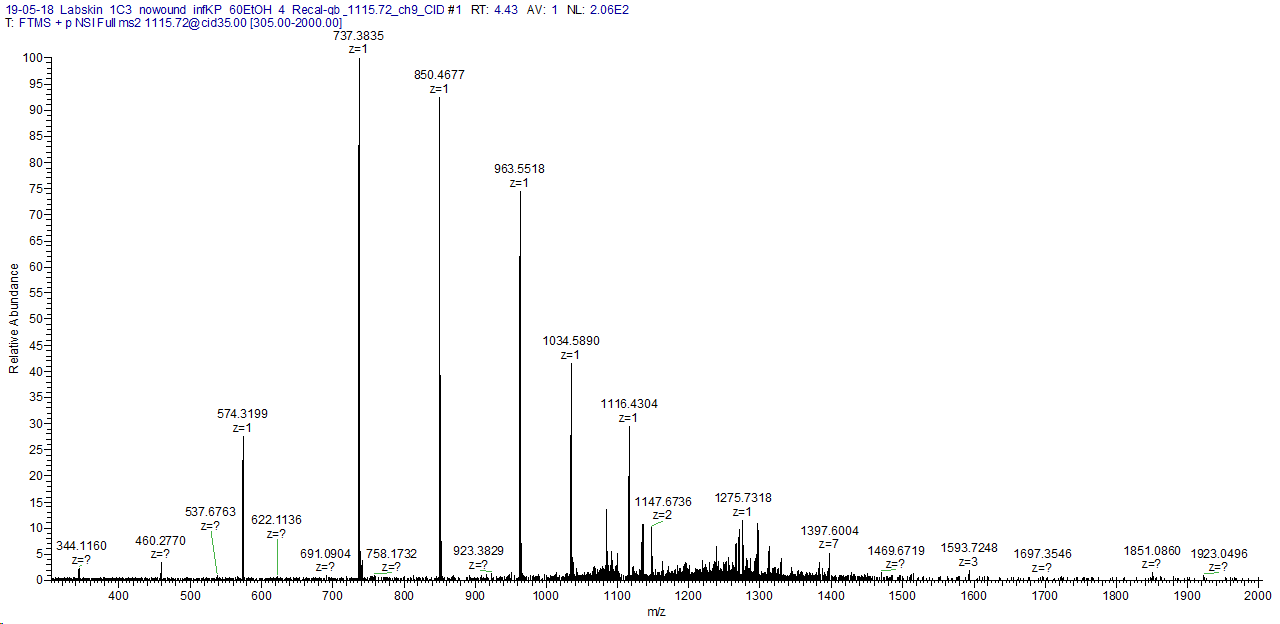


Fig. S 9 MS/MS mass spectrum of S100-A6.

Tab. S 5 List of MS/MS fragment assignments for S100-A6.

| Observed mass *m/z* | Theoretical mass *m/z* | Charge | Fragment | Mass difference [Da] | Mass difference [ppm] |
| --- | --- | --- | --- | --- | --- |
| 460.2768 | 460.2766 | 1 | y4 | 0.0002 | 0.4345 |
| 574.3199 | 574.3195 | 1 | y5 | 0.0004 | 0.6965 |
| 720.3569 | 720.3563 | 1 | y6-NH3 | 0.0006 | 0.8329 |
| 737.3834 | 737.3828 | 1 | y6 | 0.0006 | 0.8137 |
| 850.4676 | 850.4669 | 1 | y7 | 0.0007 | 0.8231 |
| 963.5517 | 963.551 | 1 | y8 | 0.0007 | 0.7265 |
| 1034.5889 | 1034.5881 | 1 | y9 | 0.0008 | 0.7733 |
| 1147.6736 | 1147.6721 | 1 | y10 | 0.0015 | 1.3070 |
| 1235.2237 | 1235.2192 | 7 | b75 | 0.0315 | 3.6431 |
| 1275.7313 | 1275.7307 | 1 | y12 | 0.0006 | 0.4703 |
| 1312.129 | 1312.1261 | 7 | b81 | 0.0203 | 2.2102 |
| 1328.2859 | 1328.2809 | 7 | b82 | 0.035 | 3.7643 |

Protein name: S100-A8

Charge state: 7+, CID

Sequence: MLTELEKALNSIIDVYHKYSLIKGNFHAVYRDDLKKLLETECPQYIRKKGADVWFKELDINTDGAVNFQEFLILVIKMGVAAHKKSHEESHKE

**
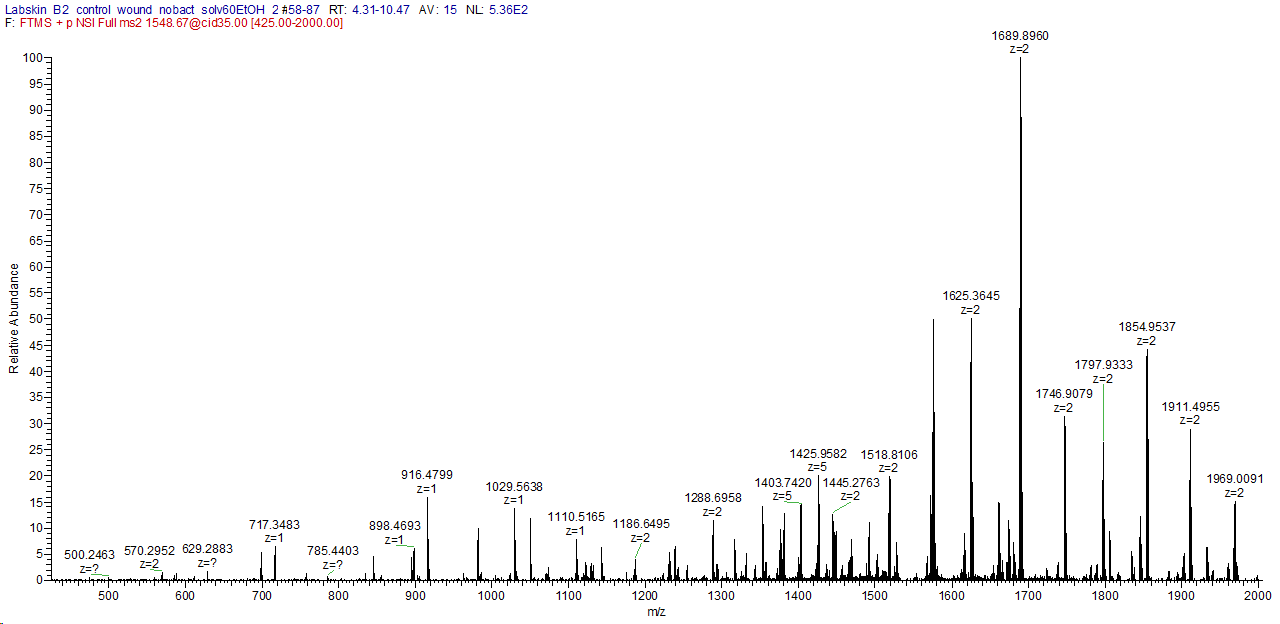
**

Fig. S 10 MS/MS mass spectrum of S100-A8.

Tab. S 6 List of MS/MS fragment assignments for S100-A8.

| Observed mass *m/z* | Theoretical mass *m/z* | Charge | Fragment | Mass error [Da] | Mass error [ppm] |
| --- | --- | --- | --- | --- | --- |
| 588.3055 | 588.3062 | 1 | b5 | -0.0007 | -1.1899 |
| 629.2883 | 629.2889 | 1 | y5 | -0.0006 | -0.9535 |
| 699.3375 | 699.3382 | 1 | b6-H2O | -0.0007 | -1.0009 |
| 717.3483 | 717.3488 | 1 | b6 | -0.0005 | -0.6970 |
| 845.4428 | 845.4437 | 1 | b7 | -0.0009 | -1.0645 |
| 895.3898 | 895.3904 | 1 | y7 | -0.0006 | -0.6701 |
| 898.4693 | 898.4703 | 1 | b8-H2O | -0.0010 | -1.1130 |
| 902.9408 | 902.9416 | 2 | y16 | -0.0016 | -0.8860 |
| 916.4799 | 916.4808 | 1 | b8 | -0.0009 | -0.9820 |
| 966.9883 | 966.9891 | 2 | y17 | -0.0016 | -0.8273 |
| 982.4214 | 982.4225 | 1 | y8 | -0.0011 | -1.1197 |
| 986.0151 | 986.0164 | 2 | b17 | -0.0026 | -1.3184 |
| 1023.5305 | 1023.5311 | 2 | y18 | -0.0012 | -0.5862 |
| 1029.5638 | 1029.5649 | 1 | b9 | -0.0011 | -1.0684 |
| 1050.0630 | 1050.0639 | 2 | b18 | -0.0018 | -0.8571 |
| 1073.0645 | 1073.0653 | 2 | y19 | -0.0016 | -0.7455 |
| 1110.5165 | 1110.5174 | 1 | y9 | -0.0009 | -0.8104 |
| 1122.5902 | 1122.5903 | 2 | b19-H2O | -0.0002 | -0.0891 |
| 1126.5829 | 1126.5813 | 1 | b10-NH3 | 0.0016 | 1.4202 |
| 1129.6064 | 1129.6073 | 2 | y20 | -0.0018 | -0.7967 |
| 1143.6069 | 1143.6078 | 1 | b10 | -0.0009 | -0.7870 |
| 1175.1095 | 1175.1116 | 2 | b20 | -0.0042 | -1.7871 |
| 1186.1483 | 1186.1494 | 2 | y21 | -0.0022 | -0.9274 |
| 1231.6506 | 1231.6536 | 2 | b21 | -0.0060 | -2.4357 |
| 1238.6110 | 1238.6124 | 1 | y10 | -0.0014 | -1.1303 |
| 1242.6909 | 1242.6914 | 2 | y22 | -0.0010 | -0.4024 |
| 1279.6824 | 1279.6824 | 2 | b22-NH3 | 0.0000 | 0.0000 |
| 1288.1940 | 1288.1956 | 2 | b22 | -0.0032 | -1.2420 |
| 1293.3352 | 1293.3364 | 3 | b33 | -0.0036 | -0.9278 |
| 1316.2244 | 1316.2256 | 2 | y23 | -0.0024 | -0.9117 |
| 1331.3104 | 1331.3108 | 5 | b56 | -0.0020 | -0.3005 |
| 1343.7224 | 1343.7239 | 1 | b12 | -0.0015 | -1.1163 |
| 1352.2413 | 1352.2431 | 2 | b23 | -0.0036 | -1.3311 |
| 1357.1204 | 1357.1194 | 5 | b57 | 0.0050 | 0.7369 |
| 1375.6702 | 1375.6713 | 1 | y11 | -0.0011 | -0.7996 |
| 1379.7358 | 1379.7362 | 5 | b58 | -0.0020 | -0.2899 |
| 1393.7414 | 1393.7373 | 5 | a59-NH3 | 0.0205 | 2.9417 |
| 1402.7396 | 1402.7416 | 5 | b59 | -0.0100 | -1.4258 |
| 1425.3564 | 1425.3584 | 5 | b60 | -0.0100 | -1.4032 |
| 1444.7749 | 1444.7762 | 2 | y25 | -0.0026 | -0.8998 |
| 1448.1655 | 1448.1670 | 5 | b61 | -0.0075 | -1.0358 |
| 1456.8064 | 1456.8080 | 1 | b13 | -0.0016 | -1.0983 |
| 1464.9732 | 1464.9712 | 5 | b62-NH3 | 0.0100 | 1.3652 |
| 1487.7855 | 1487.7798 | 5 | b63-H2O | 0.0285 | 3.8312 |
| 1491.3821 | 1491.3819 | 5 | b63 | 0.0010 | 0.1341 |
| 1502.3055 | 1502.3043 | 2 | b26-H2O | 0.0024 | 0.7988 |
| 1518.3092 | 1518.3104 | 2 | y26 | -0.0024 | -0.7904 |
| 1517.1941 | 1517.1949 | 5 | y65-H2O | -0.0040 | -0.5273 |
| 1566.3248 | 1566.3266 | 2 | y27-H2O | -0.0036 | -1.1492 |
| 1571.8330 | 1571.8349 | 1 | b14 | -0.0019 | -1.2088 |
| 1575.3296 | 1575.3319 | 2 | y27 | -0.0046 | -1.4600 |
| 1611.5238 | 1611.5280 | 3 | b41 | -0.0126 | -2.6062 |
| 1624.8634 | 1624.8661 | 2 | y28 | -0.0054 | -1.6617 |
| 1651.3737 | 1651.3793 | 2 | y29-H2O | -0.0112 | -3.3911 |
| 1660.3842 | 1660.3846 | 2 | y29 | -0.0008 | -0.2409 |
| 1673.8339 | 1673.8354 | 1 | y15 | -0.0015 | -0.8961 |
| 1679.8877 | 1679.8901 | 2 | y30-H2O | -0.0048 | -1.4287 |
| 1688.8931 | 1688.8954 | 2 | y30 | -0.0046 | -1.3618 |
| 1737.4078 | 1737.4035 | 2 | y31-H2O | 0.0086 | 2.4750 |
| 1746.4062 | 1746.4088 | 2 | y31 | -0.0052 | -1.4888 |
| 1787.9233 | 1787.9274 | 2 | y32-H2O | -0.0082 | -2.2932 |
| 1796.9300 | 1796.9327 | 2 | y32 | -0.0054 | -1.5026 |
| 1804.8740 | 1804.8759 | 1 | y16 | -0.0019 | -1.0527 |
| 1833.9653 | 1833.9667 | 1 | b16 | -0.0014 | -0.7634 |
| 1844.9484 | 1844.9489 | 2 | y33-H2O | -0.0010 | -0.2710 |
| 1853.9505 | 1853.9541 | 2 | y33 | -0.0072 | -1.9418 |
| 1881.9840 | 1881.9874 | 2 | b32 | -0.0068 | -1.8066 |
| 1901.4879 | 1901.4909 | 2 | y34-H2O | -0.0060 | -1.5777 |
| 1910.4927 | 1910.4962 | 2 | y34 | -0.0070 | -1.8320 |
| 1932.9689 | 1932.9709 | 1 | y17 | -0.0020 | -1.0347 |
| 1939.4993 | 1939.5009 | 2 | b33 | -0.0032 | -0.8250 |
| 1959.0029 | 1959.0044 | 2 | y35-H2O | -0.0030 | -0.7657 |
| 1968.0081 | 1968.0096 | 2 | y35 | -0.0030 | -0.7622 |

Protein name: S100-A7

Charge state: 7+, CID

Sequence: aSNTQAERSIIGMIDMFHKYTRRDDKIDKPSLLTMMKENFPNFLSACDKKGTNYLADVFEKKDKNEDKKIDFSEFLSLLGDIATDYHKQSHGAAPCSGGSQ

**
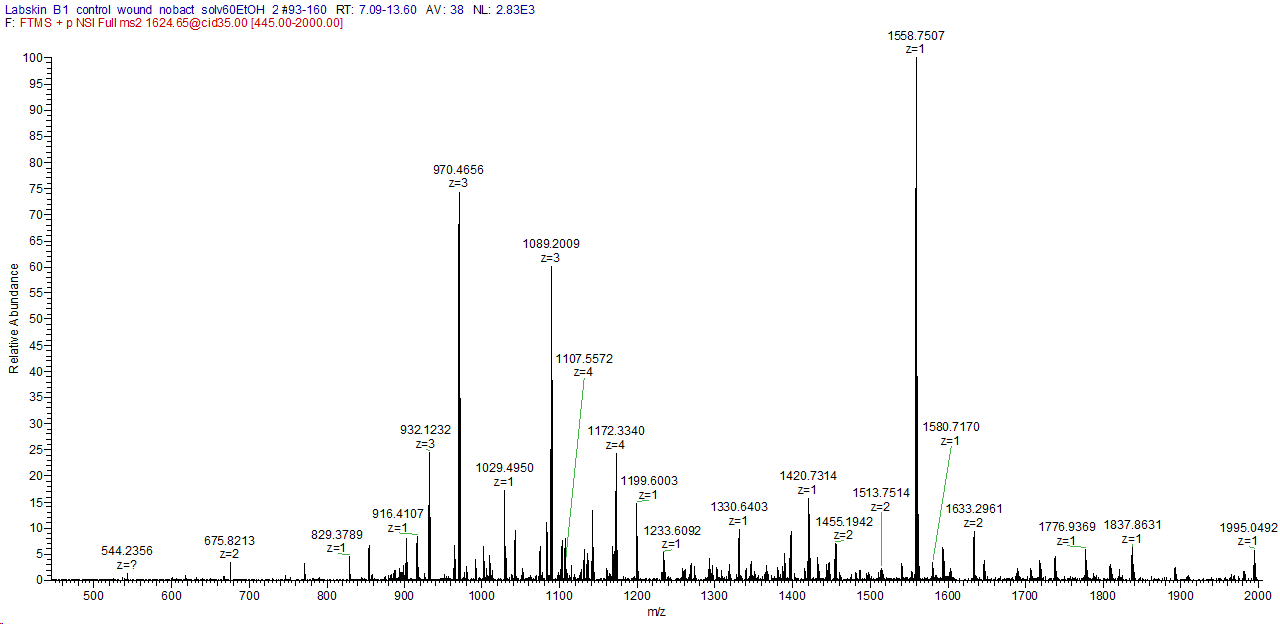
**

Fig. S 11 MS/MS mass spectrum of S100-A7.

Tab. S 7 List of MS/MS fragment assignments for S100-A7.

| Observed mass *m/z* | Theoretical mass *m/z* | Charge | Fragment | Mass error [Da] | Mass error [ppm] |
| --- | --- | --- | --- | --- | --- |
| 600.3040 | 600.3044 | 2 | b11 | -0.0008 | -0.6663 |
| 829.3789 | 829.3799 | 1 | b7 | -0.0010 | -1.2057 |
| 893.4472 | 893.4478 | 3 | b22 | -0.0018 | -0.6716 |
| 898.4001 | 898.4013 | 1 | b8-H2O | -0.0012 | -1.3357 |
| 916.4107 | 916.4119 | 1 | b8 | -0.0012 | -1.3095 |
| 925.7859 | 925.7866 | 3 | b23-H2O | -0.0021 | -0.7561 |
| 931.7890 | 931.7902 | 3 | b23 | -0.0036 | -1.2878 |
| 951.4882 | 951.4896 | 4 | b32 | -0.0056 | -1.4714 |
| 964.1285 | 964.1290 | 3 | b24-H2O | -0.0015 | -0.5186 |
| 970.1314 | 970.1325 | 3 | b24 | -0.0033 | -1.1339 |
| 976.7503 | 976.7516 | 4 | b33 | -0.0052 | -1.3309 |
| 1005.0085 | 1005.0090 | 4 | b34-H2O | -0.0020 | -0.4975 |
| 1009.5107 | 1009.5117 | 4 | b34 | -0.0040 | -0.9906 |
| 1012.8297 | 1012.8308 | 3 | b25 | -0.0033 | -1.0861 |
| 1029.4950 | 1029.4960 | 1 | b9 | -0.0010 | -0.9713 |
| 1037.7661 | 1037.7692 | 4 | b35-H2O | -0.0124 | -2.9872 |
| 1042.2708 | 1042.2718 | 4 | b35 | -0.0040 | -0.9594 |
| 1044.8549 | 1044.8500 | 3 | b26-NH3 | 0.0147 | 4.6897 |
| 1051.5101 | 1051.5115 | 2 | b18 | -0.0028 | -1.3314 |
| 1074.2942 | 1074.2955 | 4 | b36 | -0.0052 | -1.2101 |
| 1082.8630 | 1082.8643 | 3 | b27-H2O | -0.0039 | -1.2005 |
| 1088.8666 | 1088.8678 | 3 | b27 | -0.0036 | -1.1021 |
| 1102.0526 | 1102.0535 | 4 | b37-H2O | -0.0036 | -0.8167 |
| 1106.5549 | 1106.5562 | 4 | b37 | -0.0052 | -1.1748 |
| 1114.5845 | 1114.5851 | 1 | a10 | -0.0006 | -0.5383 |
| 1125.8945 | 1125.8906 | 3 | b28-NH3 | 0.0117 | 3.4639 |
| 1131.5640 | 1131.5661 | 3 | b28 | -0.0063 | -1.8558 |
| 1135.0643 | 1135.0669 | 4 | b38 | -0.0104 | -2.2906 |
| 1142.5790 | 1142.5800 | 1 | b10 | -0.0010 | -0.8752 |
| 1167.3289 | 1167.3314 | 4 | b39-H2O | -0.0100 | -2.1416 |
| 1171.8326 | 1171.8340 | 4 | b39 | -0.0056 | -1.1947 |
| 1199.6003 | 1199.6015 | 1 | b11 | -0.0012 | -1.0003 |
| 1268.3156 | 1268.3171 | 3 | b32 | -0.0045 | -1.1827 |
| 1330.6420 | 1330.6403 | 1 | b12 | 0.0017 | 1.2776 |
| 1345.6789 | 1345.6798 | 3 | b34 | -0.0027 | -0.6688 |
| 1383.3561 | 1383.3565 | 3 | b35-H2O | -0.0012 | -0.2892 |
| 1389.3584 | 1389.3600 | 3 | b35 | -0.0048 | -1.1516 |
| 1397.1814 | 1397.1816 | 2 | b23 | -0.0004 | -0.1431 |
| 1426.3889 | 1426.3828 | 3 | b36-NH3 | 0.0183 | 4.2766 |
| 1445.6883 | 1445.6898 | 2 | b24-H2O | -0.0030 | -1.0376 |
| 1454.6930 | 1454.6951 | 2 | b24 | -0.0042 | -1.4436 |
| 1558.7507 | 1558.7530 | 1 | b14 | -0.0023 | -1.4755 |
| 1632.7957 | 1632.7981 | 2 | b27 | -0.0048 | -1.4699 |
| 1689.7908 | 1689.7935 | 1 | b15 | -0.0027 | -1.5978 |

Protein name: S100-A9

Charge state: 13+, CID

Sequence: aSQLERNIETIINTFHQYSVKLGHPDTLNQGEFKELVRKDLQNFLKKENKNEKVIEHIMEDLDTNADKQLSFEEFIMLMARLTWASHEKMHEGDEGPGHHHKPGLGEGTP


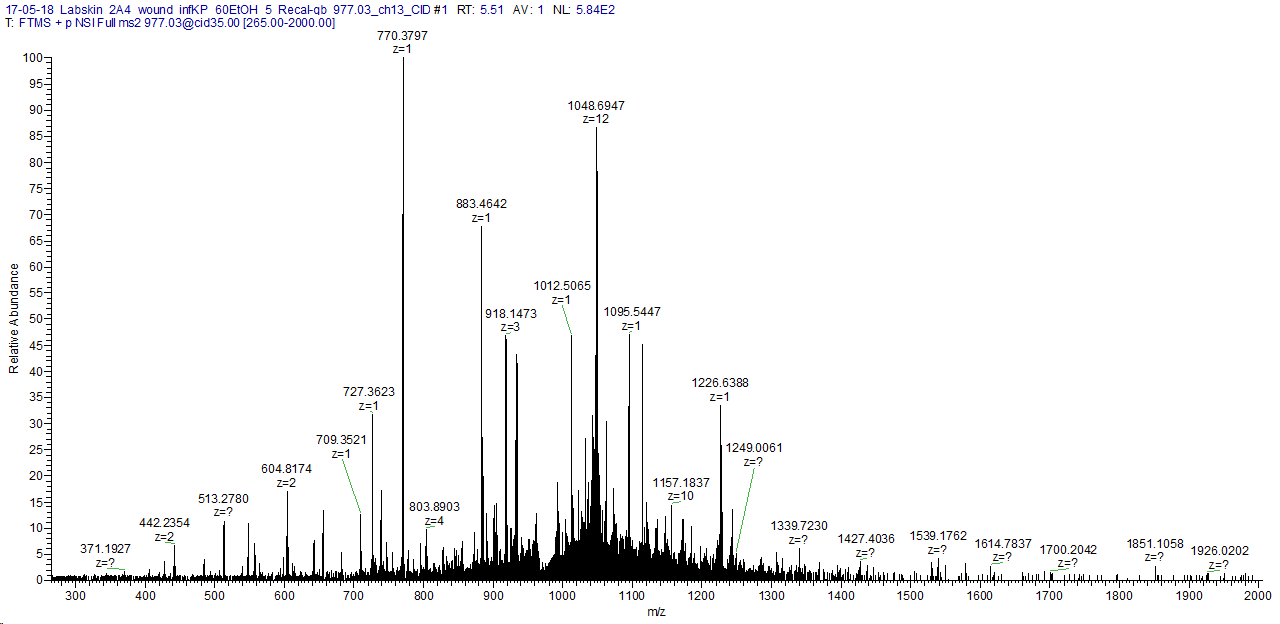


Fig. S 12 MS/MS mass spectrum of S100-A9 (CID).

Tab. S 8 List of MS/MS fragment assignments for S100-A9 (CID).

| Observed mass *m/z* | Theoretical mass *m/z* | Charge | Fragment | Mass difference [Da] | Mass difference [ppm] |
| --- | --- | --- | --- | --- | --- |
| 442.2354 | 442.2352 | 2 | b7 | 0.0004 | 0.4522 |
| 557.2805 | 557.2804 | 2 | b9 | 0.0002 | 0.1794 |
| 656.3365 | 656.3362 | 1 | b5 | 0.0003 | 0.4571 |
| 739.3689 | 739.3684 | 2 | y15 | 0.0010 | 0.6763 |
| 770.3797 | 770.3791 | 1 | b6 | 0.0006 | 0.7788 |
| 872.4136 | 872.4099 | 4 | y32 | 0.0148 | 4.2411 |
| 883.4642 | 883.4632 | 1 | b7 | 0.0010 | 1.1319 |
| 900.6791 | 900.6809 | 4 | y33 | -0.0072 | -1.9985 |
| 917.8127 | 917.8119 | 3 | b23 | 0.0024 | 0.8716 |
| 925.0856 | 925.0818 | 5 | b39 | 0.0190 | 4.1077 |
| 933.4422 | 933.4411 | 4 | y34 | 0.0044 | 1.1784 |
| 1012.5065 | 1012.5058 | 1 | b8 | 0.0007 | 0.6914 |
| 1032.1521 | 1032.1502 | 11 | y98 | 0.0209 | 1.8408 |
| 1047.0259 | 1047.0276 | 12 | y108 | -0.0204 | -1.6236 |
| 1051.2571 | 1051.2544 | 11 | b98 | 0.0297 | 2.5684 |
| 1113.5549 | 1113.5535 | 1 | b9 | 0.0014 | 1.2572 |
| 1172.3187 | 1172.3234 | 7 | b69 | -0.0329 | -4.0091 |
| 1226.6388 | 1226.6375 | 1 | b10 | 0.0013 | 1.0598 |
| 1242.6362 | 1242.6356 | 7 | b73 | 0.0042 | 0.4828 |
| 1306.006 | 1306.0031 | 6 | b66 | 0.0174 | 2.2205 |

Charge state: 16+, HCD

Sequence: MTC(S‑nitroso)KMSQLERNIETIINTFHQYSVKLGHPDTLNQGEFKELVRKDLQNFLKKENKNEKVIEHIMEDLDTNADKQLSFEEFIMLMARLTWASHEKMHEGDEGPGHHHKPGLGEGTP


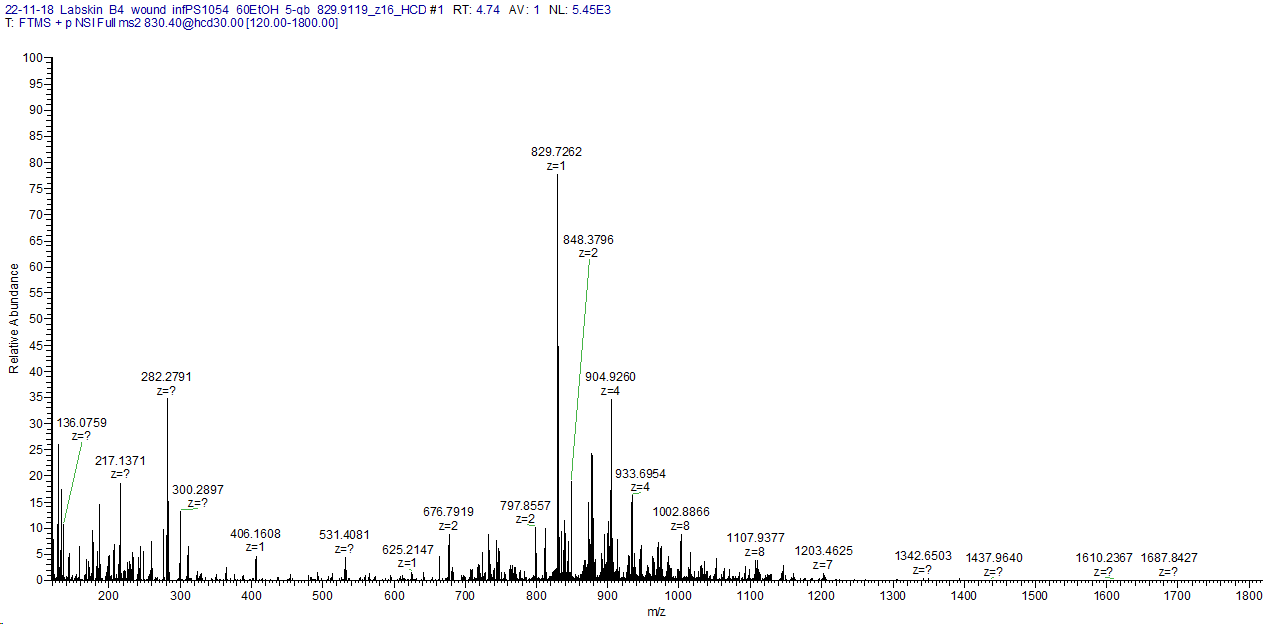


Fig. S 13 MS/MS mass spectrum of S100-A9 (HCD).

Tab. S 9 List of MS/MS fragment assignments for S100-A9 (HCD).

| Observed mass *m/z* | Theoretical mass *m/z* | Charge | Fragment | Mass difference [Da] | Mass difference [ppm] |
| --- | --- | --- | --- | --- | --- |
| 811.4214 | 811.4215 | 9 | b61 | -0.0009 | -0.1232 |
| 833.9141 | 833.9139 | 4 | b28 | 0.0008 | 0.2398 |
| 869.6997 | 869.7013 | 15 | y112 | -0.0240 | -1.8397 |
| 882.2125 | 882.2116 | 13 | y99 | 0.0117 | 1.0202 |
| 891.0102 | 891.0109 | 9 | b67 | -0.0063 | -0.7856 |
| 898.6829 | 898.6832 | 13 | y101 | -0.0039 | -0.3338 |
| 912.7241 | 912.7233 | 8 | b61 | 0.0064 | 0.8765 |
| 936.7151 | 936.7180 | 12 | y97 | -0.0348 | -3.0959 |
| 946.2240 | 946.2216 | 12 | y98 | 0.0288 | 2.5364 |
| 955.6444 | 955.6453 | 12 | y99 | -0.0108 | -0.9418 |
| 961.7126 | 961.7121 | 4 | y35 | 0.0020 | 0.5199 |
| 973.7455 | 973.7475 | 8 | b65 | -0.0160 | -2.0539 |
| 986.2777 | 986.2803 | 9 | b75 | -0.0234 | -2.6362 |
| 1002.2629 | 1002.2614 | 8 | b67 | 0.0120 | 1.4966 |
| 1014.8962 | 1014.8923 | 8 | b68 | 0.0312 | 3.8428 |
| 1029.1497 | 1029.1477 | 8 | b69 | 0.0160 | 1.9434 |
| 1032.1533 | 1032.1502 | 11 | y98 | 0.0341 | 3.0034 |
| 1038.0276 | 1038.0274 | 8 | b70 | 0.0016 | 0.1927 |
| 1042.9705 | 1042.9684 | 7 | b61 | 0.0147 | 2.0135 |
| 1052.4039 | 1052.4057 | 8 | b71 | -0.0144 | -1.7104 |
| 1059.1252 | 1059.1233 | 7 | b62 | 0.0133 | 1.7939 |
| 1084.4221 | 1084.4249 | 8 | b73 | -0.0224 | -2.5820 |
| 1098.5570 | 1098.5604 | 8 | b74 | -0.0272 | -3.0950 |
| 1109.4412 | 1109.4394 | 8 | b75 | 0.0144 | 1.6224 |
| 1121.5314 | 1121.5256 | 4 | y40 | 0.0232 | 5.1715 |
| 1128.8688 | 1128.8653 | 7 | b66 | 0.0245 | 3.1005 |
| 1145.2948 | 1145.2977 | 7 | b67 | -0.0203 | -2.5321 |
| 1159.7321 | 1159.7331 | 7 | b68 | -0.0070 | -0.8623 |
| 1202.6068 | 1202.6055 | 7 | b71 | 0.0091 | 1.0810 |

***S. aureus* NCTC13435**

Protein name: Phenol soluble modulin α-3 peptide

Charge state: 3+, CID

Sequence: fMEFVAKLFKFFKDLLGKFLGNN

**
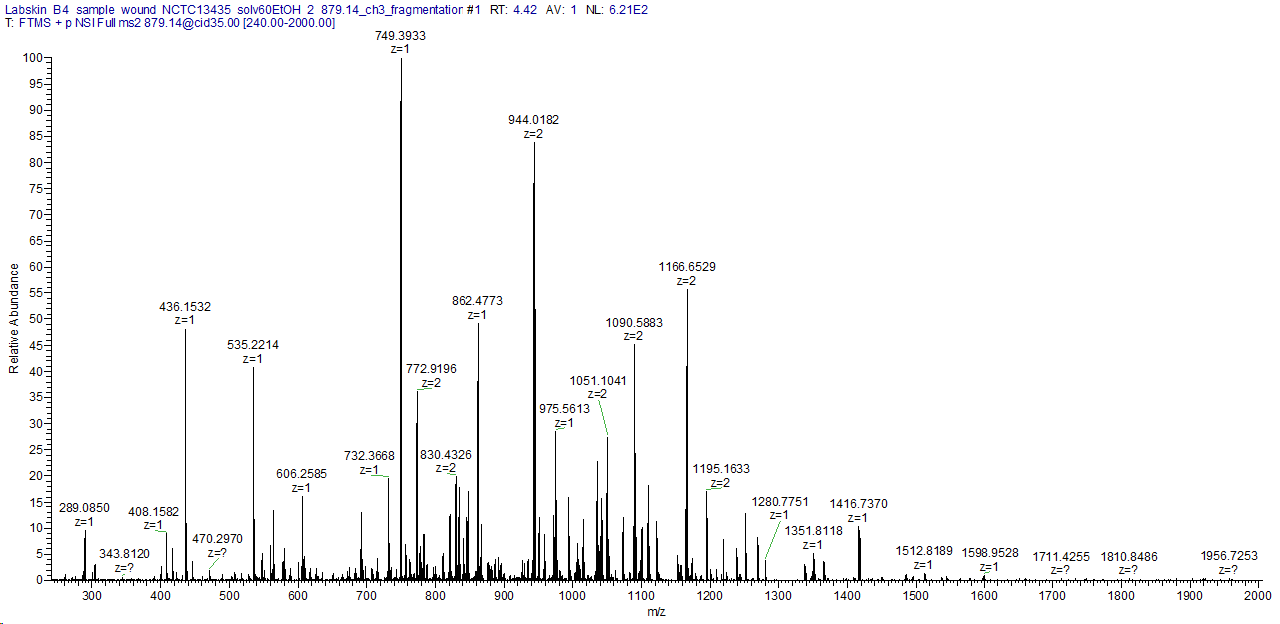
**

Fig. S 14 MS/MS mass spectrum of phenol-soluble modulin α-3 peptide (S. aureus NCTC13435).

Tab. S 10 List of MS/MS fragment assignments for phenol-soluble modulin α-3 peptide (S. aureus NCTC13435).

| Observed mass *m/z* | Theoretical mass *m/z* | Charge | Fragment | Mass error [Da] | Mass error [ppm] |
| --- | --- | --- | --- | --- | --- |
| 289.0850 | 289.0853 | 1 | b2 | -0.0003 | -1.0378 |
| 408.1582 | 408.1588 | 1 | a3 | -0.0006 | -1.4700 |
| 417.2087 | 417.2092 | 1 | y4 | -0.0005 | -1.1984 |
| 535.2214 | 535.2221 | 1 | b4 | -0.0007 | -1.3079 |
| 547.2503 | 547.2511 | 1 | y5-NH3 | -0.0008 | -1.4619 |
| 564.2770 | 564.2776 | 1 | y5 | -0.0006 | -1.0633 |
| 606.2585 | 606.2592 | 1 | b5 | -0.0007 | -1.1546 |
| 609.8450 | 609.8457 | 2 | y11 | -0.0014 | -1.1478 |
| 626.3327 | 626.3334 | 2 | b10-H2O | -0.0014 | -1.1176 |
| 635.3378 | 635.3387 | 2 | b10 | -0.0018 | -1.4166 |
| 683.3788 | 683.3799 | 2 | y12 | -0.0022 | -1.6096 |
| 692.3720 | 692.3726 | 1 | y6 | -0.0006 | -0.8666 |
| 708.8722 | 708.8729 | 2 | b11 | -0.0014 | -0.9875 |
| 716.3439 | 716.3436 | 1 | b6-H2O | 0.0003 | 0.4188 |
| 732.3668 | 732.3675 | 1 | y7-NH3 | -0.0007 | -0.9558 |
| 740.0755 | 740.076 | 3 | b18 | -0.0015 | -0.6756 |
| 748.3992 | 748.4008 | 2 | y13-NH3 | -0.0032 | -2.1379 |
| 749.3933 | 749.3941 | 1 | y7 | -0.0008 | -1.0675 |
| 756.9132 | 756.9141 | 2 | y13 | -0.0018 | -1.1890 |
| 772.9193 | 772.9203 | 2 | b12 | -0.0020 | -1.2938 |
| 777.7695 | 777.7706 | 3 | b19 | -0.0033 | -1.4143 |
| 782.7834 | 782.7839 | 3 | y20 | -0.0015 | -0.6387 |
| 796.7766 | 796.7778 | 3 | b20 | -0.0036 | -1.5061 |
| 821.4275 | 821.4285 | 2 | b13-H2O | -0.0020 | -1.2174 |
| 830.4324 | 830.4338 | 2 | b13 | -0.0028 | -1.6859 |
| 834.7906 | 834.7921 | 3 | b21 | -0.0045 | -1.7969 |
| 840.7944 | 840.7956 | 3 | b21-H2O | -0.0036 | -1.4272 |
| 847.4372 | 847.4382 | 1 | b7 | -0.0010 | -1.1800 |
| 862.4770 | 862.4781 | 1 | y8 | -0.0011 | -1.2754 |
| 886.9731 | 886.9758 | 2 | b14 | -0.0054 | -3.0441 |
| 894.4955 | 894.4958 | 2 | y15 | -0.0006 | -0.3354 |
| 934.5107 | 934.5126 | 2 | b15-H2O | -0.0038 | -2.0331 |
| 943.5165 | 943.5179 | 2 | b15 | -0.0028 | -1.4838 |
| 951.0367 | 951.0378 | 2 | y16 | -0.0022 | -1.1566 |
| 958.5339 | 958.5356 | 1 | y9-NH3 | -0.0017 | -1.7735 |
| 972.0273 | 972.0286 | 2 | b16 | -0.0026 | -1.3374 |
| 975.5609 | 975.5622 | 1 | y9 | -0.0013 | -1.3326 |
| 994.5053 | 994.5067 | 1 | b8 | -0.0014 | -1.4077 |
| 1006.0787 | 1006.08 | 2 | y17-H2O | -0.0026 | -1.2921 |
| 1015.0839 | 1015.0853 | 2 | y17 | -0.0028 | -1.3792 |
| 1036.0747 | 1036.0761 | 2 | b17 | -0.0028 | -1.3513 |
| 1041.6006 | 1041.5986 | 2 | y18-H2O | 0.0040 | 1.9201 |
| 1050.6027 | 1050.6039 | 2 | y18 | -0.0024 | -1.1422 |
| 1073.5610 | 1073.5626 | 1 | y10-NH3 | -0.0016 | -1.4904 |
| 1090.5876 | 1090.5891 | 1 | y10 | -0.0015 | -1.3754 |
| 1100.1361 | 1100.1381 | 2 | y19 | -0.0040 | -1.8180 |
| 1109.6088 | 1109.6103 | 2 | b18 | -0.0030 | -1.3518 |
| 1122.6003 | 1122.6016 | 1 | b9 | -0.0013 | -1.1580 |
| 1157.1456 | 1157.147 | 2 | b19-H2O | -0.0028 | -1.2099 |
| 1166.1508 | 1166.1523 | 2 | b19 | -0.0030 | -1.2863 |
| 1173.6710 | 1173.6723 | 2 | y20 | -0.0026 | -1.1076 |
| 1194.6613 | 1194.6631 | 2 | b20 | -0.0036 | -1.5067 |
| 1201.6556 | 1201.6575 | 1 | y11-NH3 | -0.0019 | -1.5811 |
| 1218.6828 | 1218.6841 | 1 | y11 | -0.0013 | -1.0667 |
| 1251.6852 | 1251.6845 | 2 | b21 | 0.0014 | 0.5592 |
| 1269.6680 | 1269.67 | 1 | b10 | -0.0020 | -1.5752 |
| 1365.7502 | 1365.7525 | 1 | y12 | -0.0023 | -1.6841 |
| 1416.7360 | 1416.7384 | 1 | b11 | -0.0024 | -1.6940 |
| 1659.8578 | 1659.8603 | 1 | b13 | -0.0025 | -1.5062 |

Protein name: δ-hemolysin

Charge state: 4+, CID

Sequence: fMAQDIISTIGDLVKWIIDTVNKFTKK

**
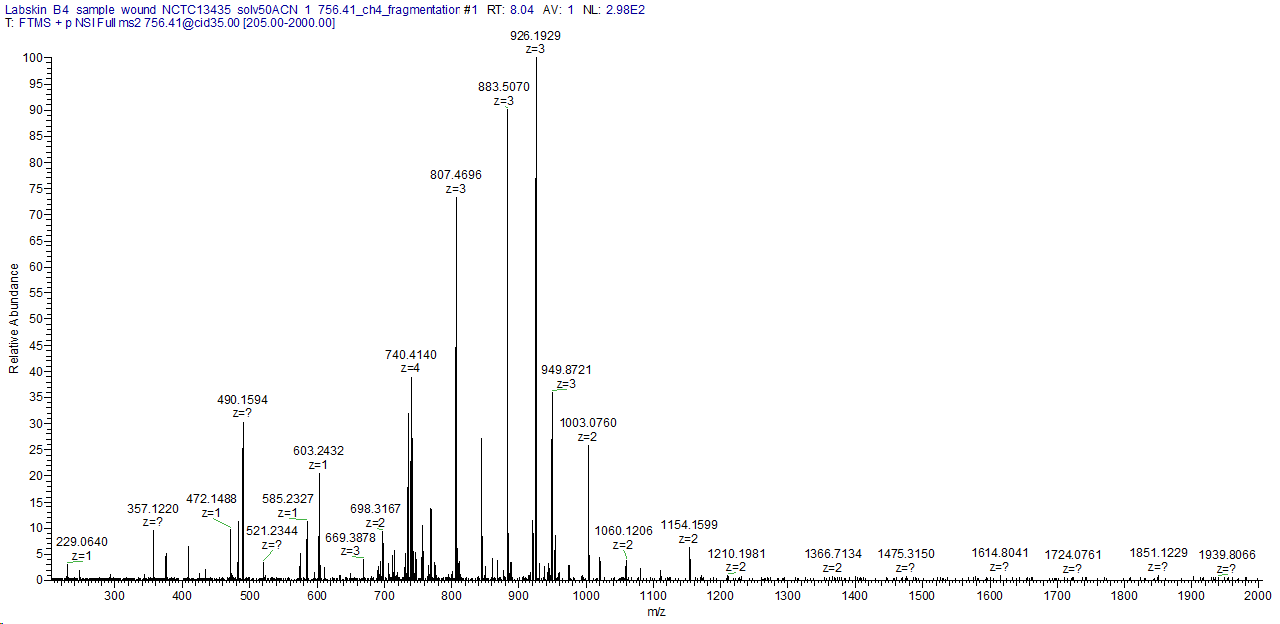
**

Fig. S 15 MS/MS mass spectrum of δ-hemolysin (S.aureus NCTC13435).

Tab. S 11 List of MS/MS fragment assignments for δ-hemolysin (S.aureus NCTC13435).

| Observed mass *m/z* | Theoretical mass *m/z* | Charge | Fragment | Mass error [Da] | Mass error [ppm] |
| --- | --- | --- | --- | --- | --- |
| 483.2916 | 483.2926 | 2 | y8 | -0.0020 | -2.0691 |
| 694.6458 | 694.6468 | 4 | y24 | -0.0040 | -1.4396 |
| 706.7484 | 706.7489 | 3 | y18 | -0.0015 | -0.7075 |
| 712.4053 | 712.4061 | 4 | y25 | -0.0032 | -1.1230 |
| 763.438 | 763.4386 | 3 | y20-H2O | -0.0018 | -0.7859 |
| 769.4409 | 769.4421 | 3 | y20 | -0.0036 | -1.5596 |
| 807.1355 | 807.1368 | 3 | y21 | -0.0039 | -1.6106 |
| 844.8302 | 844.8315 | 3 | y22 | -0.0039 | -1.5388 |
| 860.5101 | 860.5114 | 2 | y14 | -0.0026 | -1.5107 |
| 883.1725 | 883.1738 | 3 | y23 | -0.0039 | -1.4720 |
| 919.8554 | 919.8565 | 3 | y24-H2O | -0.0033 | -1.1958 |
| 925.8585 | 925.86 | 3 | y24 | -0.0045 | -1.6201 |
| 943.5343 | 943.5355 | 3 | y25-H2O | -0.0036 | -1.2718 |
| 949.5374 | 949.5391 | 3 | y25 | -0.0051 | -1.7903 |
| 953.8447 | 953.849 | 3 | b25 | -0.0129 | -4.5081 |
| 974.5653 | 974.5669 | 2 | y16 | -0.0032 | -1.6418 |
| 1003.076 | 1003.0777 | 2 | y17 | -0.0034 | -1.6948 |
| 1059.6185 | 1059.6197 | 2 | y18 | -0.0024 | -1.1325 |
| 1110.142 | 1110.1435 | 2 | y19 | -0.0030 | -1.3512 |
| 1153.6583 | 1153.6596 | 2 | y20 | -0.0026 | -1.1268 |
| 1210.1981 | 1210.2016 | 2 | y21 | -0.0070 | -2.8921 |
| 1366.2148 | 1366.2224 | 2 | b24 | -0.0152 | -5.5628 |

***S. aureus* MSSA476**

Protein name: Phenol soluble modulin α-3 peptide

Charge state: 3+, CID

Sequence: fMEFVAKLFKFFKDLLGKFLGNN

**
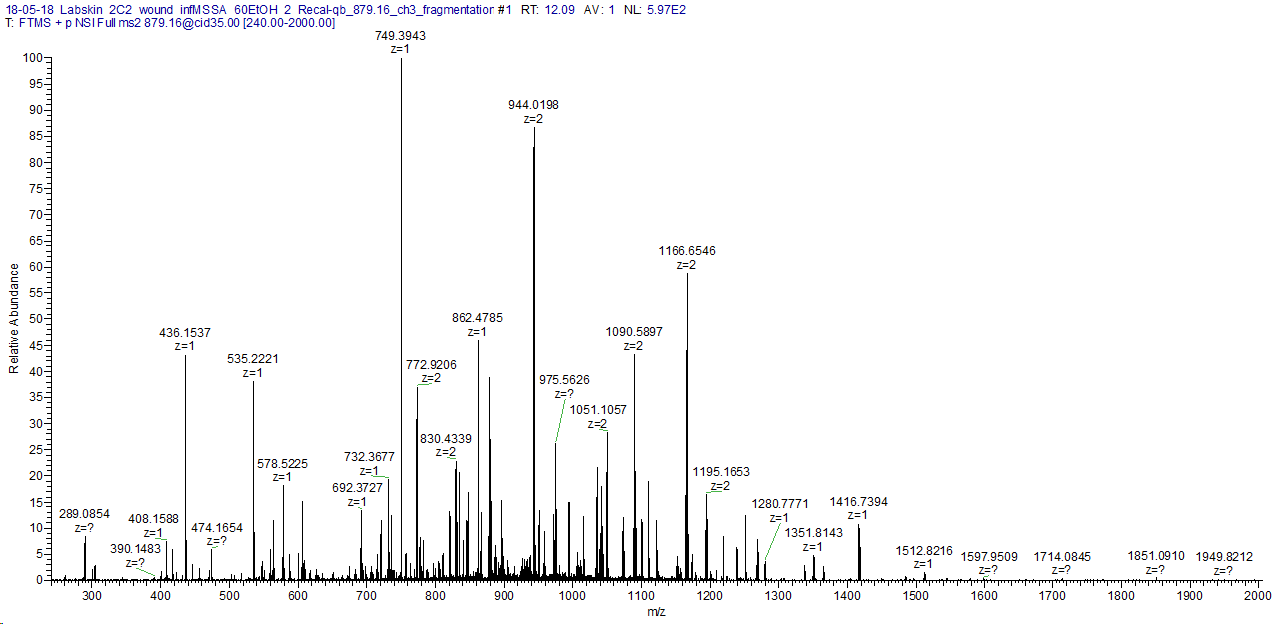
**

Fig. S 16 MS/MS mass spectrum of phenol soluble modulin α-3 peptide (S. aureus MSSA476).

Tab. S 12 List of MS/MS fragment assignments for phenol soluble modulin α-3 peptide (S. aureus MSSA476).

| Observed mass *m/z* | Theoretical mass *m/z* | Charge | Fragment | Mass difference [Da] | Mass difference [ppm] |
| --- | --- | --- | --- | --- | --- |
| 289.0854 | 289.0853 | 1 | b2 | 0.0001 | 0.3459 |
| 304.1255 | 304.1252 | 1 | y3 | 0.0003 | 0.3288 |
| 406.1537 | 406.1537 | 1 | b3 | 0.0000 | 0.2462 |
| 535.2221 | 535.2221 | 1 | b4 | 0.0000 | 0.1868 |
| 564.2777 | 564.2776 | 1 | y5 | 0.0001 | 0.1772 |
| 606.2594 | 606.2592 | 1 | b5 | 0.0002 | 0.1649 |
| 692.3727 | 692.3726 | 1 | y6 | 0.0001 | 0.1444 |
| 749.3943 | 749.3941 | 1 | y7 | 0.0002 | 0.1334 |
| 756.9142 | 756.9141 | 2 | y13 | 0.0002 | 0.0661 |
| 772.9206 | 772.9203 | 2 | b12 | 0.0006 | 0.0647 |
| 777.7716 | 777.7706 | 3 | b19 | 0.0030 | 0.0429 |
| 782.7842 | 782.7839 | 3 | y20 | 0.0009 | 0.0426 |
| 834.7925 | 834.7921 | 3 | b21 | 0.0012 | 0.0399 |
| 847.4384 | 847.4382 | 1 | b7 | 0.0002 | 0.1180 |
| 862.4785 | 862.4781 | 1 | y8 | 0.0004 | 0.1159 |
| 943.5181 | 943.5179 | 2 | b15 | 0.0004 | 0.0530 |
| 951.0382 | 951.0378 | 2 | y16 | 0.0008 | 0.0526 |
| 972.0294 | 972.0286 | 2 | b16 | 0.0016 | 0.0514 |
| 975.5626 | 975.5622 | 1 | y9 | 0.0004 | 0.1025 |
| 994.5068 | 994.5067 | 1 | b8 | 0.0001 | 0.1006 |
| 1015.0861 | 1015.0853 | 2 | y17 | 0.0016 | 0.0493 |
| 1036.0768 | 1036.0761 | 2 | b17 | 0.0014 | 0.0483 |
| 1050.6043 | 1050.6039 | 2 | y18 | 0.0008 | 0.0476 |
| 1090.5897 | 1090.5891 | 1 | y10 | 0.0006 | 0.0917 |
| 1100.1385 | 1100.1381 | 2 | y19 | 0.0008 | 0.0454 |
| 1109.6108 | 1109.6103 | 2 | b18 | 0.0010 | 0.0451 |
| 1122.6024 | 1122.6016 | 1 | b9 | 0.0008 | 0.0891 |
| 1166.1528 | 1166.1523 | 2 | b19 | 0.0010 | 0.0429 |
| 1194.664 | 1194.6631 | 2 | b20 | 0.0018 | 0.0419 |
| 1218.6842 | 1218.6841 | 1 | y11 | 0.0001 | 0.0821 |
| 1251.6855 | 1251.6845 | 2 | b21 | 0.0020 | 0.0399 |
| 1269.6707 | 1269.6700 | 1 | b10 | 0.0007 | 0.0788 |
| 1365.7532 | 1365.7525 | 1 | y12 | 0.0007 | 0.0732 |
| 1416.7394 | 1416.7384 | 1 | b11 | 0.0010 | 0.0706 |
| 1512.8216 | 1512.8209 | 1 | y13 | 0.0007 | 0.0661 |

Protein name: δ-hemolysin

Charge state: 4+, CID

Sequence: fMAQDIISTISDLVKWIIDTVNKFTKK


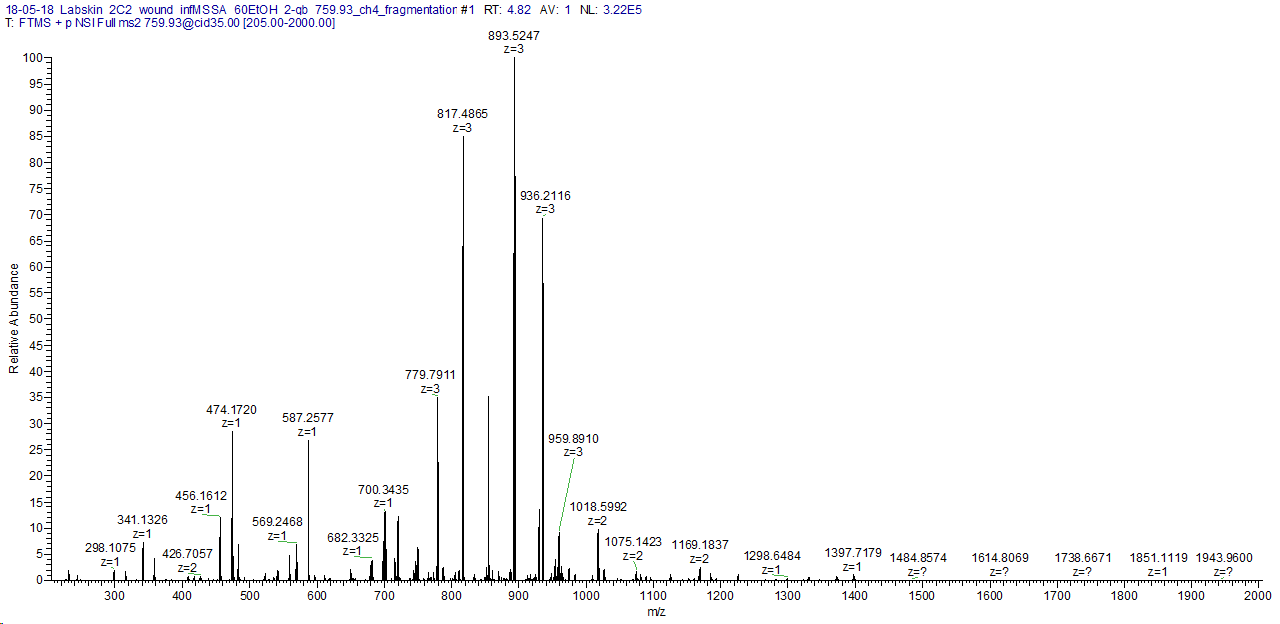


Fig. S 17 MS/MS mass spectrum of δ-hemolysin (S. aureus MSSA476).

Tab. S 13 List of MS/MS fragment assignments for δ-hemolysin (S. aureus MSSA476).

| Observed mass *m/z* | Theoretical mass *m/z* | Charge | Fragment | Mass difference [Da] | Mass difference [ppm] |
| --- | --- | --- | --- | --- | --- |
| 231.0800 | 231.0798 | 1 | b2 | 0.0002 | 0.8655 |
| 359.1358 | 359.1384 | 1 | b3 | -0.0026 | 0.5569 |
| 383.2347 | 383.2345 | 2 | y6 | 0.0004 | 0.2609 |
| 432.7686 | 432.7687 | 2 | y7 | -0.0002 | 0.2311 |
| 474.1654 | 474.1653 | 1 | b4 | 0.0001 | 0.4218 |
| 483.2927 | 483.2926 | 2 | y8 | 0.0002 | 0.2069 |
| 523.3239 | 523.3239 | 1 | y4 | 0.0000 | 0.3822 |
| 574.0101 | 574.0101 | 3 | y14 | 0.0000 | 0.1161 |
| 587.2496 | 587.2494 | 1 | b5 | 0.0002 | 0.3406 |
| 597.3482 | 597.3481 | 2 | y10 | 0.0002 | 0.1674 |
| 611.7049 | 611.7047 | 3 | y15 | 0.0006 | 0.1090 |
| 650.0472 | 650.0471 | 3 | y16 | 0.0003 | 0.1026 |
| 653.8905 | 653.8901 | 2 | y11 | 0.0008 | 0.1529 |
| 679.0580 | 679.0577 | 3 | y17 | 0.0009 | 0.0982 |
| 700.3339 | 700.3334 | 1 | b6 | 0.0005 | 0.2856 |
| 702.1498 | 702.1495 | 4 | y24 | 0.0012 | 0.0712 |
| 719.9091 | 719.9088 | 4 | y25 | 0.0012 | 0.0695 |
| 723.1416 | 723.1412 | 4 | b25 | 0.0016 | 0.0691 |
| 746.9301 | 746.9298 | 2 | y12 | 0.0006 | 0.1339 |
| 779.4462 | 779.4457 | 3 | y20 | 0.0015 | 0.0855 |
| 787.3658 | 787.3655 | 1 | b7 | 0.0003 | 0.2540 |
| 817.1409 | 817.1403 | 3 | y21 | 0.0018 | 0.0816 |
| 854.8354 | 854.8350 | 3 | y22 | 0.0012 | 0.0780 |
| 860.5119 | 860.5114 | 2 | y14 | 0.0010 | 0.1162 |
| 893.1780 | 893.1773 | 3 | y23 | 0.0021 | 0.0746 |
| 935.8642 | 935.8635 | 3 | y24 | 0.0021 | 0.0712 |
| 959.5436 | 959.5426 | 3 | y25 | 0.0030 | 0.0695 |
| 974.5679 | 974.5669 | 2 | y16 | 0.0020 | 0.1026 |
| 1018.0839 | 1018.0830 | 2 | y17 | 0.0018 | 0.0982 |
| 1074.6256 | 1074.6250 | 2 | y18 | 0.0012 | 0.0931 |
| 1080.6055 | 1080.6048 | 1 | y9 | 0.0007 | 0.1851 |
| 1088.5320 | 1088.5292 | 1 | b10 | 0.0028 | 0.1837 |
| 1125.1496 | 1125.1488 | 2 | y19 | 0.0016 | 0.0889 |
| 1168.6655 | 1168.6648 | 2 | y20 | 0.0014 | 0.0856 |
| 1193.6899 | 1193.6888 | 1 | y10 | 0.0011 | 0.1675 |
| 1225.2078 | 1225.2069 | 2 | y21 | 0.0018 | 0.0816 |
| 1281.7509 | 1281.7489 | 2 | y22 | 0.0040 | 0.0780 |
| 1415.7102 | 1415.7087 | 1 | b13 | 0.0015 | 0.1413 |

***K. pneumoniae* KP257**

Protein name: Uncharacterized protein (gene KPN_00497, Uniprot accession no. A6T5S6)

Charge state: 9+, CID

Sequence: AQLITKEEVKHFKLTKVGPISVGPSGGEFSSPSDLHDQLSKLADEKGGKYYVITAAREHGPNFEATAEVYK


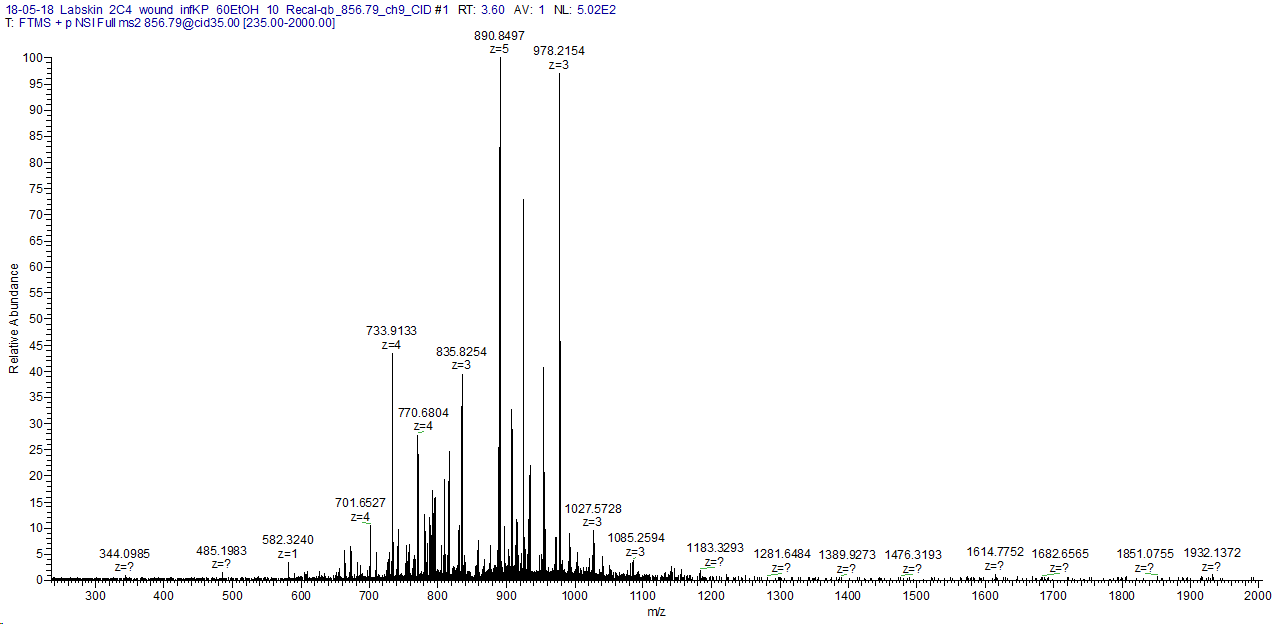


Fig. S 18 MS/MS mass spectrum of uncharacterized protein (gene KPN_00497, Uniprot accession no. A6T5S6).

Tab. S 14 List of MS/MS fragment assignments for uncharacterized protein (gene KPN_00497, Uniprot accession no. A6T5S6).

| Observed mass *m/z* | Theoretical mass *m/z* | Charge | Fragment | Mass difference [Da] | Mass difference [ppm] |
| --- | --- | --- | --- | --- | --- |
| 663.3220 | 663.3222 | 2 | y12 | -0.0004 | -0.3015 |
| 672.8912 | 672.8916 | 4 | b25 | -0.0016 | -0.5944 |
| 687.1468 | 687.1470 | 4 | b26 | -0.0008 | -0.2911 |
| 701.4017 | 701.4024 | 4 | b27 | -0.0028 | -0.9980 |
| 733.6627 | 733.6630 | 4 | b28 | -0.0012 | -0.4089 |
| 742.2078 | 742.2087 | 6 | y40 | -0.0054 | -1.2126 |
| 757.8837 | 757.8845 | 4 | y27 | -0.0032 | -1.0556 |
| 770.4297 | 770.4301 | 4 | b29 | -0.0016 | -0.5192 |
| 783.4615 | 783.4615 | 3 | b21 | 0.0000 | 0.0000 |
| 792.1875 | 792.1881 | 4 | b30 | -0.0024 | -0.7574 |
| 795.7296 | 795.7308 | 6 | y43 | -0.0072 | -1.5080 |
| 816.4836 | 816.4843 | 3 | b22 | -0.0021 | -0.8573 |
| 830.6256 | 830.6266 | 5 | y37 | -0.0050 | -1.2039 |
| 835.4908 | 835.4915 | 3 | b23 | -0.0021 | -0.8378 |
| 876.4287 | 876.4294 | 6 | y49 | -0.0042 | -0.7987 |
| 890.4485 | 890.4490 | 5 | y40 | -0.0025 | -0.5615 |
| 896.8527 | 896.8531 | 3 | b25 | -0.0012 | -0.4460 |
| 907.8550 | 907.8554 | 5 | y41 | -0.0020 | -0.4406 |
| 915.8595 | 915.8602 | 3 | b26 | -0.0021 | -0.7643 |
| 925.2609 | 925.2618 | 5 | y42 | -0.0045 | -0.9727 |
| 934.8668 | 934.8674 | 3 | b27 | -0.0018 | -0.6418 |
| 954.6753 | 954.6755 | 5 | y43 | -0.0010 | -0.2095 |
| 977.8811 | 977.8816 | 3 | b28 | -0.0015 | -0.5113 |
| 1003.2921 | 1003.2926 | 5 | y46 | -0.0025 | -0.4984 |
| 1026.9036 | 1026.9044 | 3 | b29 | -0.0024 | -0.7790 |
| 1040.1075 | 1040.1095 | 5 | y48 | -0.0100 | -1.9229 |

Protein name: DNA-binding protein HU-α

Charge state: 11+, CID

Sequence: MNKTQLIDVIADKADLSKAQAKAALESTLAAITESLKEGDAVQLVGFGTFKVNHRAERTGRNPQTGKEIKIAAANVPAFVSGKALKDAVK


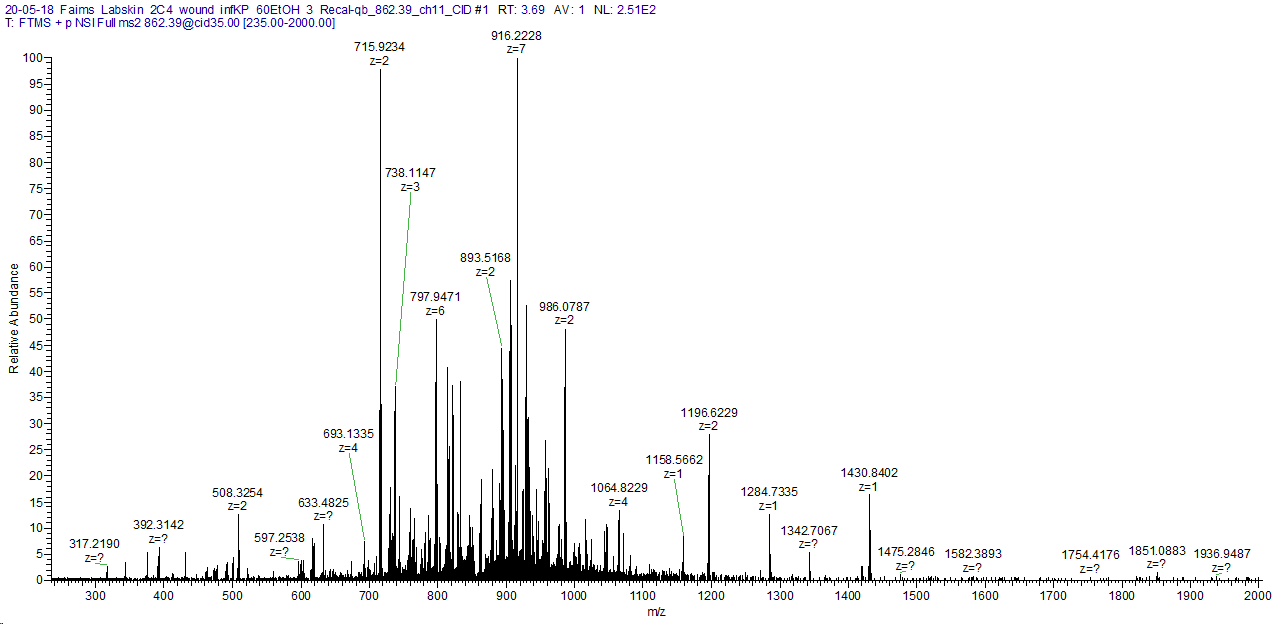


Fig. S 19 MS/MS mass spectrum of DNA-binding protein HU-α.

Tab. S 15 List of MS/MS fragment assignments for DNA-binding protein HU-α.

| Observed mass *m/z* | Theoretical mass *m/z* | Charge | Fragment | Mass difference [Da] | Mass difference [ppm] |
| --- | --- | --- | --- | --- | --- |
| 317.2190 | 317.2183 | 1 | y3 | 0.0007 | 2.2067 |
| 432.2461 | 432.2453 | 1 | y4 | 0.0008 | 1.8508 |
| 673.4255 | 673.4243 | 1 | y6 | 0.0012 | 1.7819 |
| 692.6325 | 692.6310 | 4 | b26 | 0.0060 | 2.1657 |
| 715.9234 | 715.9219 | 2 | y14 | 0.0030 | 2.0952 |
| 737.7804 | 737.7790 | 3 | y22 | 0.0042 | 1.8976 |
| 744.4628 | 744.4614 | 1 | y7 | 0.0014 | 1.8806 |
| 765.4579 | 765.4561 | 2 | y15 | 0.0036 | 2.3515 |
| 785.6842 | 785.6812 | 4 | b30 | 0.0120 | 3.8183 |
| 787.9400 | 787.9405 | 6 | y44 | -0.0030 | -0.6346 |
| 797.4453 | 797.4441 | 6 | y45 | 0.0072 | 1.5048 |
| 813.9572 | 813.9555 | 6 | y46 | 0.0102 | 2.0886 |
| 822.4791 | 822.4776 | 2 | y16 | 0.0030 | 1.8238 |
| 828.9367 | 828.9349 | 2 | b15 | 0.0036 | 2.1715 |
| 832.8059 | 832.8028 | 6 | y47 | 0.0186 | 3.7224 |
| 846.4700 | 846.4701 | 7 | y56 | -0.0007 | -0.1181 |
| 879.3417 | 879.3401 | 7 | y58 | 0.0112 | 1.8195 |
| 885.4785 | 885.4769 | 2 | b16 | 0.0032 | 1.8069 |
| 895.4965 | 895.4950 | 7 | y59 | 0.0105 | 1.6751 |
| 905.6448 | 905.6431 | 7 | y60 | 0.0119 | 1.8771 |
| 915.7934 | 915.7913 | 7 | y61 | 0.0147 | 2.2931 |
| 929.0348 | 929.0333 | 2 | y19 | 0.0030 | 1.6146 |
| 931.9471 | 931.9462 | 7 | y62 | 0.0063 | 0.9657 |
| 956.7343 | 956.7315 | 5 | y45 | 0.0140 | 2.9266 |
| 958.8165 | 958.8147 | 7 | y64 | 0.0126 | 1.8773 |
| 961.3092 | 961.3066 | 9 | y83 | 0.0234 | 2.7047 |
| 976.5473 | 976.5451 | 5 | y46 | 0.0110 | 2.2528 |
| 985.5775 | 985.5753 | 2 | y20 | 0.0044 | 2.2322 |
| 1016.6117 | 1016.6099 | 1 | y10 | 0.0018 | 1.7706 |
| 1023.5612 | 1023.5602 | 3 | b29 | 0.0030 | 0.9770 |
| 1043.5589 | 1043.5554 | 1 | b9 | 0.0035 | 3.3539 |
| 1046.5634 | 1046.5596 | 4 | b40 | 0.0152 | 3.6309 |
| 1064.3213 | 1064.3189 | 4 | b41 | 0.0096 | 2.2550 |
| 1070.9203 | 1070.9183 | 3 | b31 | 0.0060 | 1.8676 |
| 1342.7067 | 1342.7035 | 1 | b12 | 0.0032 | 2.3833 |
| 1430.8402 | 1430.8366 | 1 | y14 | 0.0036 | 2.5160 |

***P. aeruginosa* PS1054**

Protein name: DNA-binding protein HU-β

Charge state: 11+, CID

Sequence: MNKSELIDAIAASADIPKAVAGRALDAVIESVTGALKAGDSVVLVGFGTFAVKERAARTGRNPQTGKPIKIAAAKIPGFKAGKALKDAVN

**
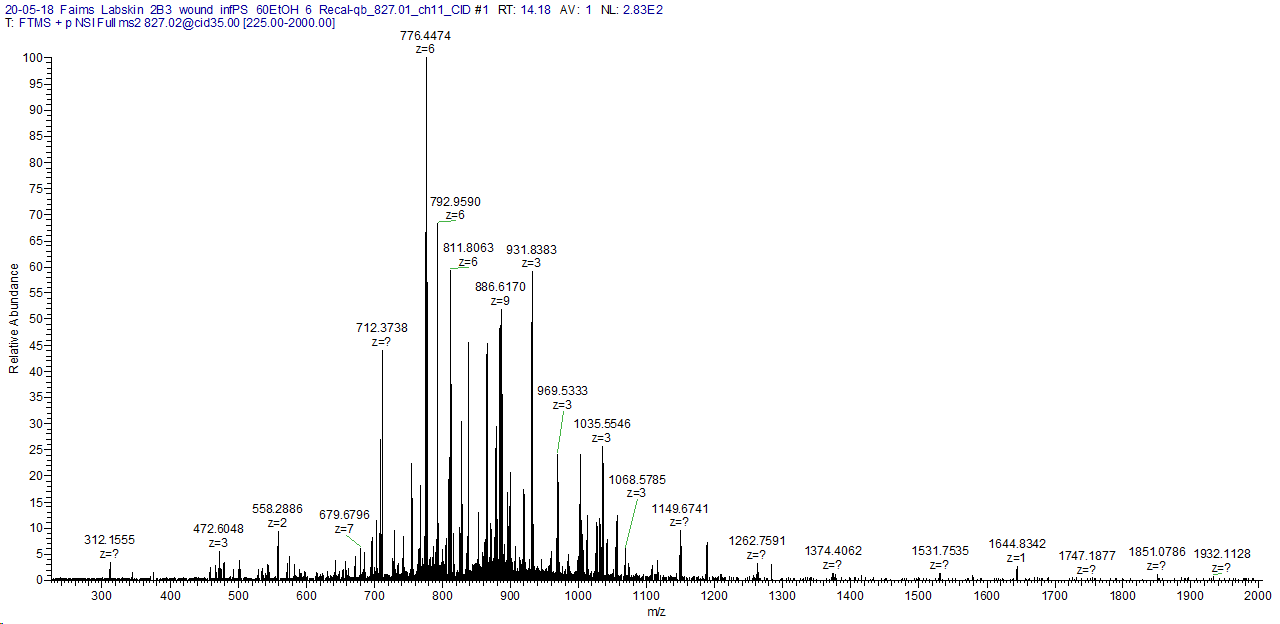
**

Fig. S 20 MS/MS mass spectrum of DNA-binding protein HU-β.

Tab. S 16 List of MS/MS fragment assignments for DNA-binding protein HU-β.

| Observed mass *m/z* | Theoretical mass *m/z* | Charge | Fragment | Mass difference [Da] | Mass difference [ppm] |
| --- | --- | --- | --- | --- | --- |
| 472.6048 | 472.6050 | 3 | y14 | -0.0006 | -0.4232 |
| 679.5373 | 679.5363 | 7 | y46 | 0.0070 | 1.4716 |
| 703.3443 | 703.3443 | 1 | b6 | 0.0000 | 0.0000 |
| 709.8438 | 709.8438 | 7 | y48 | 0.0000 | 0.0000 |
| 730.4093 | 730.4094 | 1 | y7 | -0.0001 | -0.1369 |
| 733.6706 | 733.6732 | 8 | y58 | -0.0208 | -3.5438 |
| 742.0976 | 742.0981 | 6 | y43 | -0.0030 | -0.6738 |
| 764.9459 | 764.9459 | 2 | y15 | 0.0000 | 0.0000 |
| 776.1131 | 776.1131 | 6 | y45 | 0.0000 | 0.0000 |
| 811.4720 | 811.4719 | 6 | y47 | 0.0006 | 0.1232 |
| 838.3399 | 838.3397 | 7 | y58 | 0.0014 | 0.2386 |
| 859.4926 | 859.4924 | 7 | y59 | 0.0014 | 0.2327 |
| 859.0006 | 859.0000 | 6 | y50 | 0.0036 | 0.6985 |
| 864.9256 | 864.9255 | 7 | y60 | 0.0007 | 0.1156 |
| 870.3824 | 870.3852 | 9 | y78 | -0.0252 | -3.2170 |
| 878.2783 | 878.2782 | 9 | b87 | 0.0009 | 0.1139 |
| 883.3595 | 883.3602 | 7 | y61 | -0.0049 | -0.7924 |
| 886.1714 | 886.1712 | 9 | y80 | 0.0018 | 0.2257 |
| 896.0079 | 896.0077 | 10 | y89 | 0.0020 | 0.2232 |
| 899.5161 | 899.5150 | 7 | y62 | 0.0077 | 1.2229 |
| 919.4035 | 919.4099 | 9 | y83 | -0.0576 | -6.9610 |
| 931.5038 | 931.5040 | 3 | b28 | -0.0006 | -0.2147 |
| 959.2887 | 959.2867 | 8 | b76 | 0.0160 | 2.0849 |
| 969.1986 | 969.1987 | 3 | b29 | -0.0003 | -0.1032 |
| 1006.0375 | 1006.0373 | 4 | b41 | 0.0008 | 0.1988 |
| 1012.2126 | 1012.2129 | 3 | b30 | -0.0009 | -0.2964 |
| 1030.8038 | 1030.8044 | 4 | b42 | -0.0024 | -0.5821 |
| 1041.2237 | 1041.2236 | 3 | b31 | 0.0003 | 0.0960 |
| 1055.5713 | 1055.5715 | 4 | b43 | -0.0008 | -0.1895 |
| 1107.9314 | 1107.9289 | 3 | b33 | 0.0075 | 2.2565 |
| 1115.5769 | 1115.5765 | 1 | b10 | 0.0004 | 0.3586 |
| 1150.6162 | 1150.6151 | 3 | b35 | 0.0033 | 0.9560 |
| 1188.3099 | 1188.3098 | 3 | b36 | 0.0003 | 0.0842 |

Protein name: Uncharacterized protein (gene PA4739, Uniprot accession no. Q9HV60)

Charge state: 9+, HCD

Sequence: ANDTMQKTEEAVSDTWITSKVKSSLIANKNVSGVDIKVETNKGVVSLSGNVKSDAERDLAIETAKGIKGVKAVSADGLKSVE


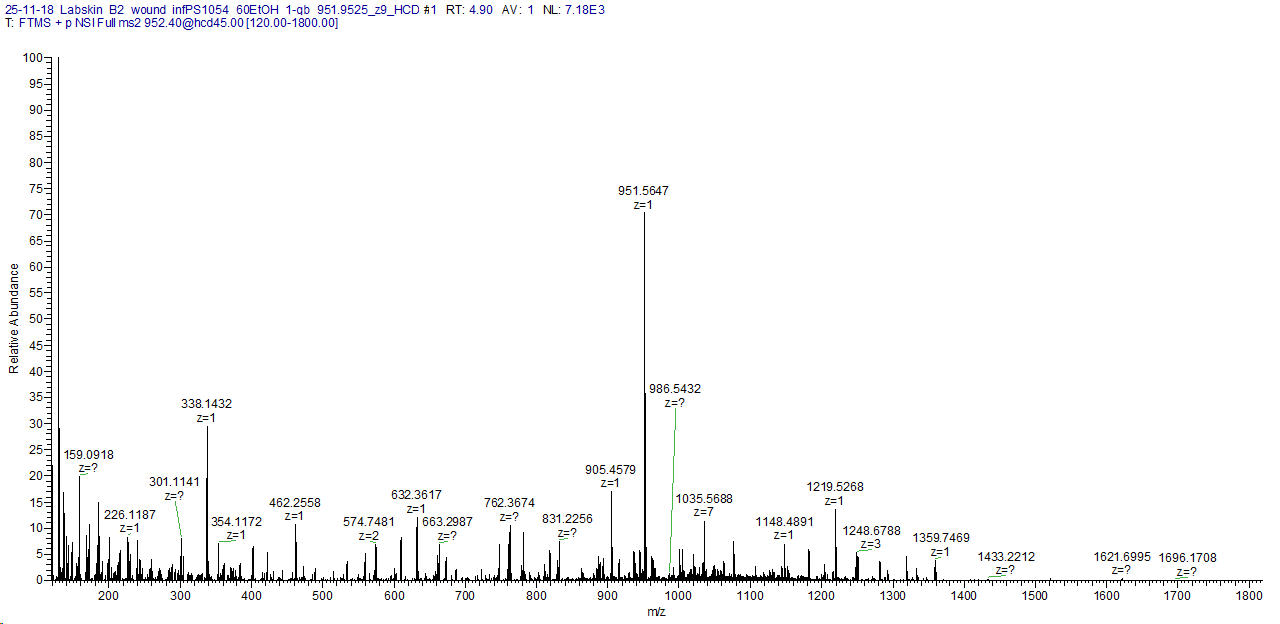


Fig. S 21 MS/MS mass spectrum of uncharacterized protein (gene PA4739, Uniprot accession no. Q9HV60).

Tab. S 17 List of MS/MS fragment assignments for uncharacterized protein (gene PA4739, Uniprot accession no. Q9HV60).

| Observed mass *m/z* | Theoretical mass *m/z* | Charge | Fragment | Mass difference [Da] | Mass difference [ppm] |
| --- | --- | --- | --- | --- | --- |
| 402.1617 | 402.1619 | 1 | b4 | -0.0002 | -0.4973 |
| 462.2558 | 462.2558 | 1 | y4 | 0.0000 | 0.0000 |
| 574.7481 | 574.7481 | 2 | b10 | 0.0000 | 0.0000 |
| 610.2668 | 610.2666 | 2 | b11 | 0.0004 | 0.3277 |
| 632.3614 | 632.3617 | 1 | y6 | -0.0003 | -0.4744 |
| 661.2613 | 661.2610 | 1 | b6 | 0.0003 | 0.4537 |
| 680.3778 | 680.3775 | 2 | y14 | 0.0006 | 0.4409 |
| 714.6516 | 714.6514 | 4 | y28 | 0.0008 | 0.2799 |
| 747.3890 | 747.3883 | 1 | y7 | 0.0007 | 0.9366 |
| 789.3562 | 789.3560 | 2 | b7 | 0.0004 | 0.2534 |
| 797.1895 | 797.1899 | 4 | y31 | -0.0016 | -0.5018 |
| 802.6152 | 802.6148 | 6 | y47 | 0.0024 | 0.4984 |
| 811.3545 | 811.3541 | 2 | b15 | 0.0008 | 0.4930 |
| 818.4260 | 818.4254 | 1 | y8 | 0.0006 | 0.7331 |
| 864.7235 | 864.7231 | 4 | y34 | 0.0016 | 0.4626 |
| 886.4811 | 886.4811 | 4 | y35 | 0.0000 | 0.0000 |
| 890.4035 | 890.4036 | 1 | b8 | -0.0001 | -0.1123 |
| 914.7004 | 914.7005 | 5 | y45 | -0.0005 | -0.1093 |
| 936.5104 | 936.5101 | 4 | y37 | 0.0012 | 0.3203 |
| 961.2763 | 961.2772 | 4 | y38 | -0.0036 | -0.9363 |
| 962.9354 | 962.9363 | 5 | y47 | -0.0045 | -0.9346 |
| 965.3976 | 965.3979 | 7 | y66 | -0.0021 | -0.3108 |
| 991.9791 | 991.9807 | 7 | y67 | -0.0112 | -1.6129 |
| 1000.2982 | 1000.2997 | 4 | y40 | -0.0060 | -1.4996 |
| 1004.5258 | 1004.5259 | 1 | y10 | -0.0001 | -0.0995 |
| 1006.4153 | 1006.4161 | 7 | y68 | -0.0056 | -0.7949 |
| 1019.4463 | 1019.4462 | 1 | b9 | 0.0001 | 0.0981 |
| 1019.8858 | 1019.8857 | 3 | y30 | 0.0003 | 0.0981 |
| 1022.8474 | 1022.8485 | 7 | y69 | -0.0077 | -1.0754 |
| 1034.5665 | 1034.5661 | 5 | y51 | 0.0020 | 0.3866 |
| 1035.2812 | 1035.2816 | 7 | y70 | -0.0028 | -0.3864 |
| 1054.3810 | 1054.3798 | 5 | y52 | 0.0060 | 1.1381 |
| 1062.5839 | 1062.5840 | 3 | y31 | -0.0003 | -0.0941 |
| 1075.5632 | 1075.5630 | 1 | y11 | 0.0002 | 0.1859 |
| 1077.1882 | 1077.1883 | 5 | y53 | -0.0005 | -0.0928 |
| 1107.2813 | 1107.2824 | 6 | y65 | -0.0066 | -0.9934 |
| 1118.3568 | 1118.3567 | 4 | y44 | 0.0004 | 0.0894 |
| 1133.6237 | 1133.6212 | 3 | y33 | 0.0075 | 2.2053 |
| 1143.1242 | 1143.1238 | 4 | y45 | 0.0016 | 0.3499 |
| 1148.4891 | 1148.4888 | 1 | b10 | 0.0003 | 0.2612 |
| 1152.6307 | 1152.6283 | 3 | y34 | 0.0072 | 2.0822 |
| 1152.6307 | 1152.6283 | 3 | y34 | 0.0072 | 2.0822 |
| 1175.1481 | 1175.1475 | 4 | y46 | 0.0024 | 0.5106 |
| 1181.6393 | 1181.6390 | 3 | y35 | 0.0009 | 0.2539 |
| 1203.6595 | 1203.6579 | 1 | y12 | 0.0016 | 1.3293 |
| 1219.5268 | 1219.5259 | 1 | b11 | 0.0009 | 0.7380 |
| 1248.3438 | 1248.3444 | 3 | y37 | -0.0018 | -0.4806 |
| 1250.2941 | 1250.2948 | 3 | b35 | -0.0021 | -0.5599 |
| 1281.3661 | 1281.3672 | 3 | y38 | -0.0033 | -0.8585 |
| 1292.9544 | 1292.9558 | 4 | y51 | -0.0056 | -1.0828 |
| 1318.5943 | 1318.5944 | 1 | b12 | -0.0001 | -0.0758 |
| 1333.3969 | 1333.3971 | 3 | y40 | -0.0006 | -0.1500 |
| 1359.7469 | 1359.7478 | 1 | y14 | -0.0009 | -0.6619 |
| 1520.6523 | 1520.6533 | 1 | b14 | -0.0010 | -0.6576 |
| 1621.6995 | 1621.7010 | 1 | b15 | -0.0015 | -0.9250 |

Protein name: Uncharacterized protein (gene PA0039, Uniprot accession no. Q9I793)

Charge state: 5+, HCD

Sequence: AKPCEELKAEIDAKIKANGVPAYTLEIVDKGSVTDKKVVGTCDGGTKEIVYQRG


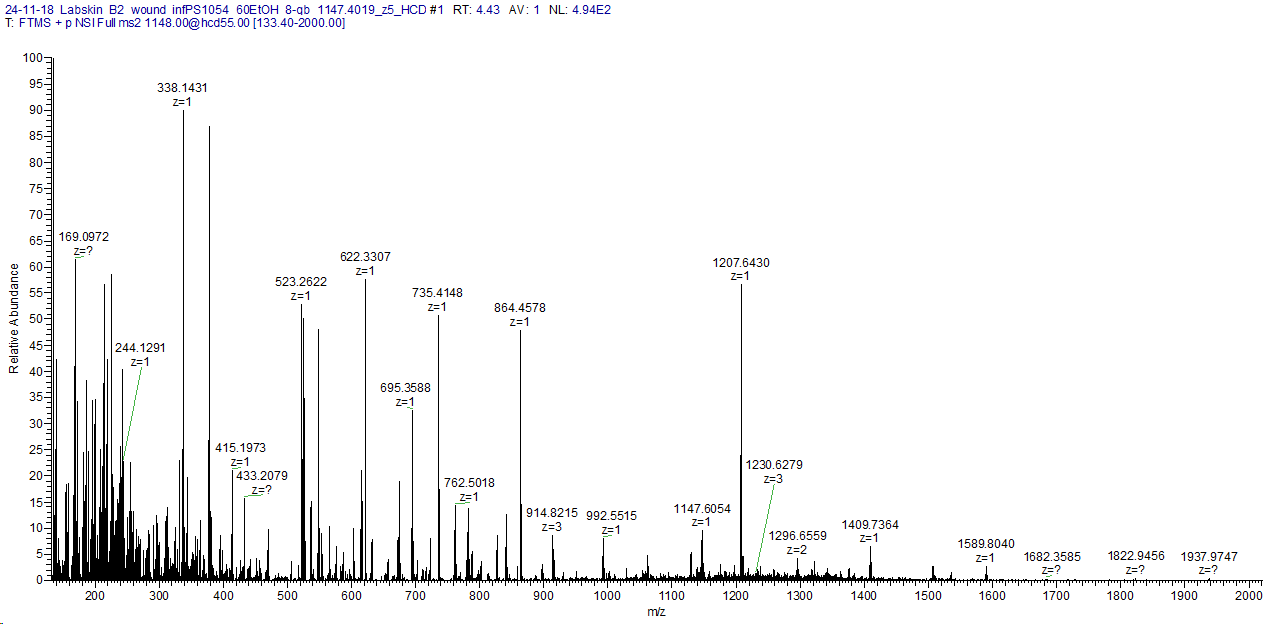


*Fig. S 22 MS/MS mass spectrum of uncharacterized protein (gene PA0039, Uniprot accession no. Q9I793).*

Tab. S 18 List of MS/MS fragment assignments for uncharacterized protein (gene PA0039, Uniprot accession no. Q9I793).

| Observed mass *m/z* | Theoretical mass *m/z* | Charge | Fragment | Mass difference [Da] | Mass difference [ppm] |
| --- | --- | --- | --- | --- | --- |
| 523.2625 | 523.2623 | 1 | y4 | 0.0002 | 0.3822 |
| 604.3256 | 604.3251 | 2 | y11 | 0.0010 | 0.1655 |
| 622.3312 | 622.3307 | 1 | y5 | 0.0005 | 0.3214 |
| 735.4154 | 735.4148 | 1 | y6 | 0.0006 | 0.2720 |
| 864.4583 | 864.4574 | 1 | y7 | 0.0009 | 0.2314 |
| 992.5531 | 992.5524 | 1 | y8 | 0.0007 | 0.2015 |
| 1207.6430 | 1207.6430 | 1 | y11 | 0.0000 | 0.1656 |
| 1218.1350 | 1218.1402 | 4 | b47 | -0.0206 | -4.2360 |
| 360.1985 | 360.1990 | 1 | y3 | -0.0005 | -1.3881 |

**References**

1. Wisplinghoff, H., 181 - Pseudomonas spp., Acinetobacter spp. and Miscellaneous Gram-Negative Bacilli. In *Infectious Diseases (Fourth Edition)*, Cohen, J.; Powderly, W. G.; Opal, S. M., Eds. Elsevier: 2017; pp 1579-1599.

2. Otter, J. A.; Yezli, S.; French, G. L., The Role of Contaminated Surfaces in the Transmission of Nosocomial Pathogens. In *Use of Biocidal Surfaces for Reduction of Healthcare Acquired Infections*, Borkow, G., Ed. Springer International Publishing: Cham, 2014; pp 27-58.
